# The PTPN1/N2 Peptide Inhibitor Achieves Potent, Long-Acting Glycemic Control and Weight-Loss Dual Efficacy in Mice

**DOI:** 10.1021/acsomega.6c05930

**Published:** 2026-09-23

**Authors:** Yiying Shi, Xianmin Yang, Fanxu Meng, Anton Feenstra, Jin Chen, Zongwen Gu, Xiang Gao, Wenqing Yao, Guozhen Dong, Liyan Gong, Han Wu, Chuanliang Zhang, Yuchao Gu

**Affiliations:** † Qingdao Key Laboratory of Biomacromolecular Drug Discovery and Development, College of Chemical Engineering, 66280 Qingdao University of Science and Technology, Qingdao 266042, China; ‡ Qingdao Center of Technology Innovation for Shark Antibody Development, College of Biological Engineering, Qingdao University of Science and Technology, Qingdao 266042, China; § Department of Computer Science, 1190 Vrije Universiteit Amsterdam, 1081HV Amsterdam, The Netherlands; ∥ School of Medicine and Pharmacy, Ocean University of China, Qingdao 266003, China

## Abstract

Type 2 diabetes mellitus and obesity are closely linked metabolic disorders driven by insulin and leptin resistance. Current long-acting therapies capable of addressing both conditions are dominated by incretin-based agents, which primarily achieve efficacy through appetite suppression and are frequently associated with tolerability limitations and metabolic rebound after treatment discontinuation. Alternative strategies that correct underlying signaling defects without suppressing food intake remain lacking. Protein tyrosine phosphatases PTPN1 and PTPN2 act as coordinated intracellular negative regulators of insulin and leptin signaling by dephosphorylating key molecules within the IRS-AKT and JAK2-STAT3 pathways. Although genetic studies have established these phosphatases as attractive therapeutic targets, translation has been hindered by the absence of long-acting inhibitors with robust *in vivo* efficacy. Here we report the rational design of **Y5**, a chemically engineered BimBH3-derived peptide analogue that functions as a dual inhibitor of PTPN1 and PTPN2. Fatty-acid conjugation and hapten modification confer high enzymatic potency, enhanced proteolytic stability, strong albumin binding, and prolonged half-life and systemic exposure. **Y5** restores cellular insulin and leptin signaling *in vitro*, leading to coordinated activation of IRS-1, AKT, and STAT3 under resistant conditions. In diabetic db/db mice, **Y5** once-weekly administration induces sustained glycemic control, improves glucose tolerance, and reduces circulating insulin levels, consistent with enhanced insulin sensitivity. In DIO mice, **Y5** induces pronounced and sustained weight loss while preserving food intake, demonstrating a potential mechanism distinct from incretin-based therapies. **Y5** selectively reduces adiposity, improves hepatic steatosis, and is well tolerated across dosing regimens. These findings establish long-acting dual inhibition of PTPN1 and PTPN2 as a mechanistically distinct therapeutic strategy for T2DM and obesity. By reversing hormone resistance rather than suppressing appetite, **Y5** defines an alternative paradigm for achieving long-acting glycemic control and weight loss *in vivo*.

## Graphical abstract

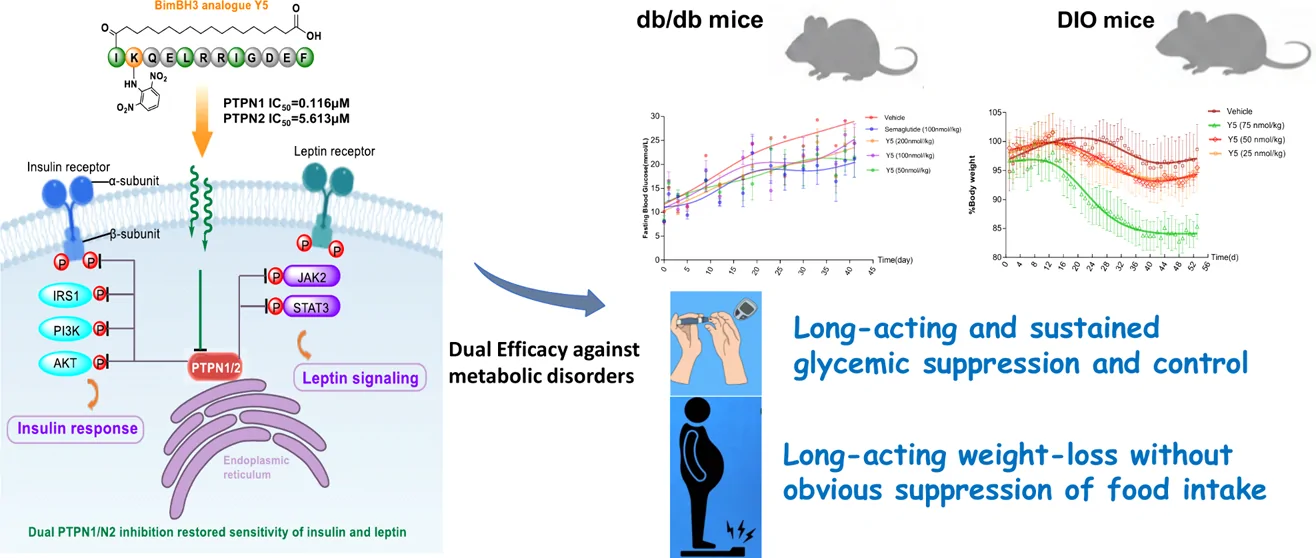

## Introduction

Type 2 diabetes mellitus (T2DM) and obesity are tightly interconnected metabolic disorders whose global prevalence continues to rise despite substantial advances in pharmacotherapy.^1–3^ Although lifestyle interventions, such as increased physical activity, strict dietary regimens, and existing pharmacotherapies, have shown limited long-term efficacy, many patients struggle to manage T2DM and/or obesity.^4^ In recent years, incretin-based agents, particularly glucagon-like peptide-1 receptor (GLP-1R) agonists and related multiagonists, have emerged as the dominant clinical approach capable of delivering both glycemic control and weight loss.^5–13^ However, these therapies achieve efficacy primarily through sustained suppression of appetite, a mechanism intrinsically associated with gastrointestinal adverse effects, diminished eating-related quality of life, and clinically significant metabolic rebound following treatment discontinuation.^14–17^ In addition, a meta-analysis revealed that discontinuation of GLP-1 receptor agonists could lead to consistent and clinically meaningful rebound effects across multiple cardiometabolic parameters, including body weight, waist circumference, glycemic control, and blood pressure.^18^ The near-exclusive reliance on appetite-driven strategies highlights a fundamental limitation of current therapies and underscores the need for alternative approaches capable of correcting metabolic dysfunction independently of caloric intake reduction. Consequently, there emerges a new and unmet subdivided clinical need for new weight-loss pharmacotherapies capable of delivering robust, clinically meaningful weight loss without suffering from gastrointestinal adverse effects, affecting appetite, or requiring dietary adjustments.

Insulin and leptin resistance represent central pathophysiological drivers linking hyperglycaemia and excess adiposity. At the intracellular level, protein tyrosine phosphatases PTPN1 (also known as PTP1B) and PTPN2 (also known as TC-PTP) function as coordinated negative regulators of insulin and leptin signaling by dephosphorylating key components of the insulin receptor, IRS-AKT and JAK2-STAT3 pathways,^19^ including direct negative regulation of insulin and leptin signaling,^20–23^ modulation of hypothalamic energy homeostasis and glucose metabolism,^23,24^ as well as T-cell based immune responses.^24–27^ These physiological studies suggested the potential for the inhibition of PTPN1/2 for the treatment of obesity and T2DM via improving and/or reversing insulin and leptin resistance.^19,22,28–33^ And theoretically, it will not cause the aforementioned side effects such as nausea, vomiting, and appetite loss.

Insulin resistance is a common feature of type 2 diabetes and obesity. PTPN1 is a negative regulator of both insulin and leptin signaling to dephosphorylate the activated insulin receptor (IR) and/or insulin receptor substrates (IRS)^34–37^ and was generally regarded as a promising therapeutic target for metabolic diseases such as diabetes and obesity.^38,39^ PTPN2 also could contribute to the regulation of insulin receptor signaling by catalyzing insulin receptor dephosphorylation to limit the duration of insulin signaling. PTPN1/2 functioned in a coordinated manner to achieve an overall regulation of phosphorylation and signaling of insulin receptor.^22^ Moreover, PTPN1/2 exert their inhibitory effects on leptin signaling by inducing the dephosphorylation of JAK2 ^40^ and STAT3,^41^ respectively.

Despite compelling biological validation, translation of PTPN1/N2 inhibition into effective therapies has remained elusive. Small-molecule inhibitors targeting the highly conserved and polar catalytic pockets of phosphatases have demonstrated limited cellular efficacy, poor selectivity, and unfavorable pharmacokinetic properties, precluding potent target engagement *in vivo*.^19,42^ Consequently, no PTPN1/PTPN2-directed agents have previously achieved potent long-acting glycemic control or meaningful weight loss in animal models of metabolic disease. Thus, the effective modulation of intracellular phosphatases may require a fundamentally different molecular strategy capable of achieving prolonged, tissue-relevant inhibition rather than transient enzymatic blockade.^19,43^

Here we report a long-acting peptide-based strategy that overcomes these translational barriers by enabling dual inhibition of PTPN1 and PTPN2 with potent long-acting glycemic control and weight loss dual efficacy in mice. By rationally engineering a BimBH3-derived peptide through fatty-acid conjugation and hapten modification, we generated **Y5** (Figure 1a), a dual PTPN1/PTPN2 inhibitor with exceptional proteolytic stability, prolonged systemic exposure, and favorable tissue distribution. We demonstrate that **Y5** restores insulin and leptin signaling, achieves potent long-acting glycemic control, and induces pronounced weight loss indicated by unchanged food intake in multiple mouse models of metabolic disease. These findings establish dual PTPN1/PTPN2 inhibition as a mechanistically distinct and therapeutically viable alternative to incretin-based approaches.

**Figure 1:**
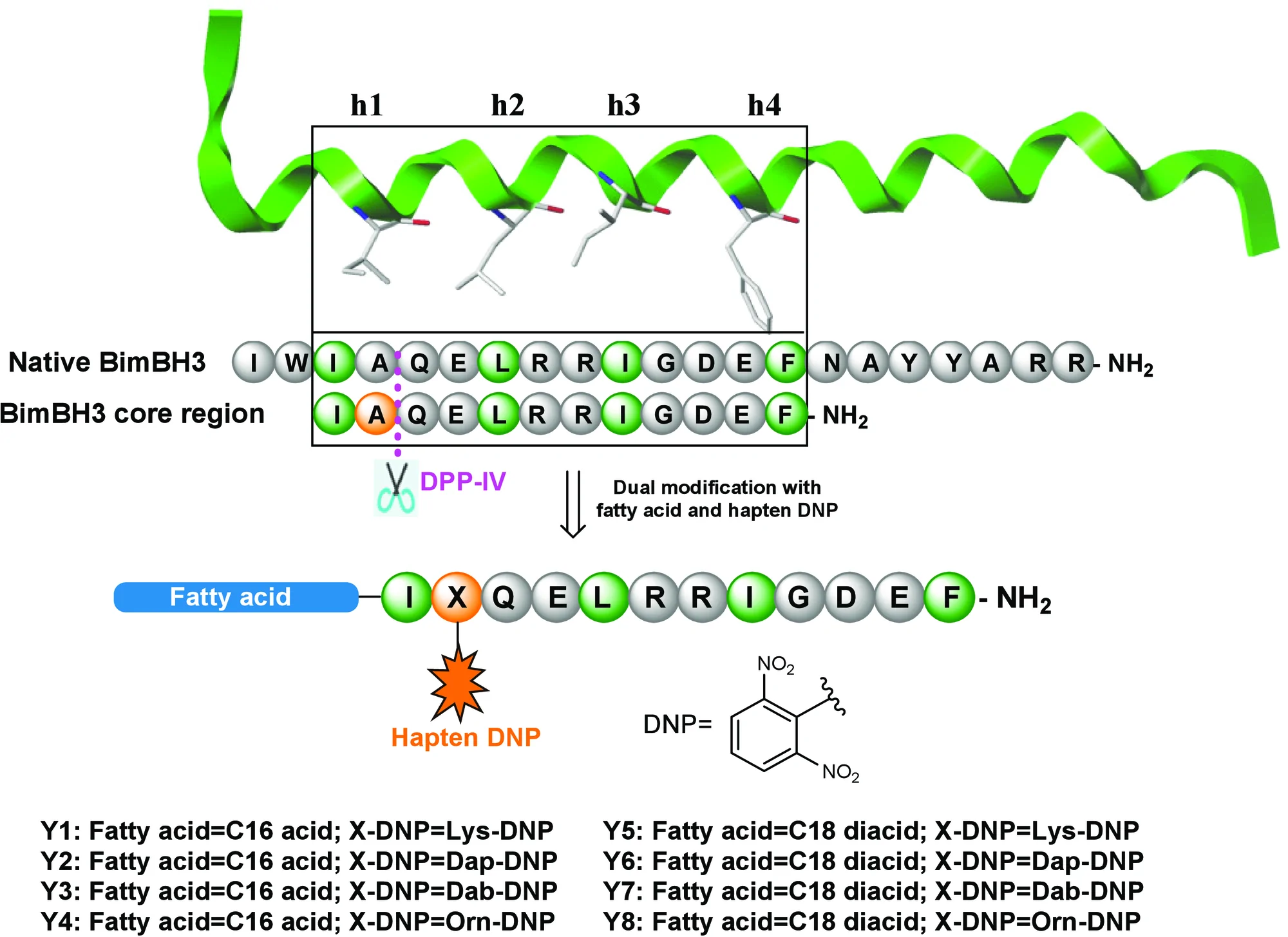
Sequence of core region of native BimBH3 and the design of the novel BimBH3 analogues with dual modifications with fatty acid and hapten DNP.

## Results

### Design and Synthesis of Dual-Targeting Long-Acting Peptide Inhibitors

Given the coordinated roles of PTPN1 and PTPN2 in attenuating insulin and leptin signaling, we reasoned that durable correction of metabolic dysfunction would require simultaneous inhibition of both phosphatases. To address this challenge, we explored a peptide-based strategy capable of engaging the conserved phosphatase active sites while enabling prolonged systemic exposure. We selected the BH3 domain of the pro-apoptotic protein Bim as a starting scaffold based on its established ability to engage protein–protein interaction interfaces and its previously reported activity against PTPN1. Importantly, we subsequently found that this scaffold, after appropriate chemical modification, also displays inhibitory potential against PTPN2, thereby establishing the feasibility of designing dual PTPN1/PTPN2 inhibitors.

To achieve long-acting dual PTPN1/PTPN2 inhibition, we systematically engineered the BimBH3 core sequence through two complementary modifications: (i) fatty acid conjugation to promote high-affinity serum albumin binding, a well-established strategy for extending the plasma half-life of peptide therapeutics; and (ii) hapten (DNP) modification to enhance molecular stability and systemic persistence. Notably, we further leveraged an antibody-recruitment strategy. Endogenous antibodies against small-molecule haptens naturally exist in human serum, constituting 1–8% of total antibodies. By conjugating the DNP hapten to the peptide, these peptide analogues can bind to these pre-existing antibodies, forming immune complexes that benefit from FcRn-mediated recycling via the antibody Fc fragment, thereby potentially contributing to prolonged half-life via a mechanism that warrants future investigation. This design not only overcomes the pharmacokinetic and intracellular target-engagement limitations that have constrained prior phosphatase-directed approaches, but also achieves a dual half-life extension mechanism (albumin binding plus antibody recruitment) while reducing immunogenicity risks by utilizing the natural antibody repertoire.

Based on this rationale, we designed and synthesized series of analogues (Y1–Y8) by varying two structural parameters: the N-terminal fatty acid type (C16 acid vs C18 diacid) and the length of the DNP hapten linker attached to the Lys^2^ side chain (Lys, Dap, Dab, Orn). These modifications were systematically investigated for their effects on PTPN1/PTPN2 inhibitory activity, metabolic stability, and long-acting biological performance.

The designed BimBH3 analogues (Figure 1) were synthesized via Fmoc-based solid-phase peptide synthesis (SPPS) using 2-chlorotrityl chloride (2-Cl-CTC) resin, followed by standard cleavage protocols to yield the target peptides (Scheme 1). Postsynthesis, crude products underwent purification through preparative reversed-phase HPLC equipped with C18 columns. Final compound integrity was verified through dual analytical characterization: molecular masses were confirmed by electrospray ionization mass spectrometry (ESI-MS), while purity levels (>95% in all cases) were determined by analytical HPLC. Full synthetic validation data, including chromatograms (Figures S1–S9) and mass spectral analyses (Table S1), are provided in the Supporting Information.

**Scheme 1:**
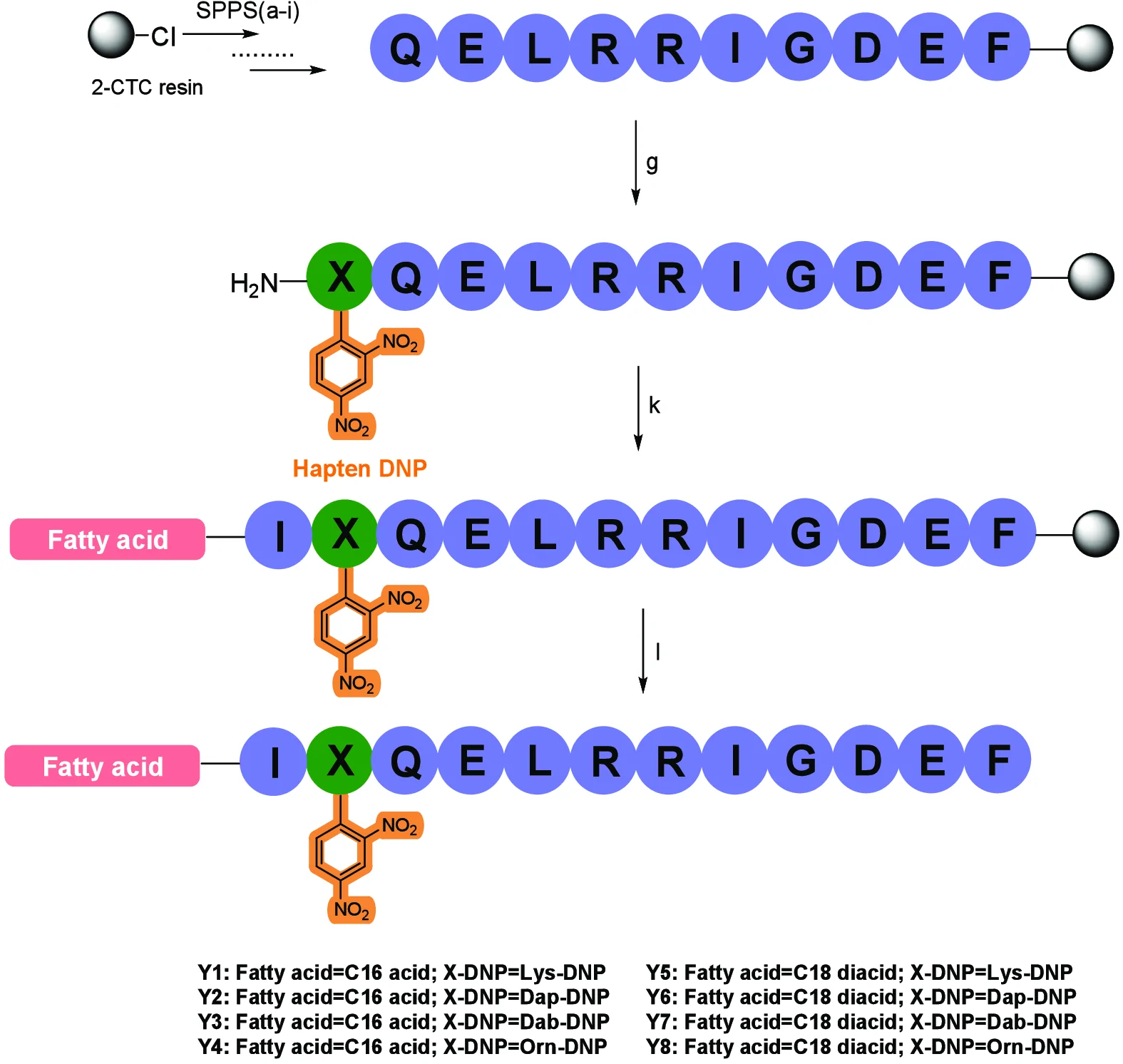
General Solid Phase Syntheses of BimBH3 Analogues with Dual Fatty Acid Conjugation^a^

a Reagents and conditions: a. (1) Fmoc-Phe-OH, DIEA, DCM, rt; (2) 20% piperidine, DMF, 40 °C; b–i. (1) Fmoc protected amino acids, HOBt, DIC, DMF, 40 °C; (2) 20% piperidine, DMF, 40 °C; g. (1) 20% piperidine, DMF, 40 °C; (2) Fmoc-X­(DNP)-OH, HOBt, HBTU, DIEA, 40 °C, 1 h; (3) X = Lys, Dab, Dap, Orn; k. (1) 20% piperidine, DMF, 40 °C; (2) Fmoc-Ile-OH, HOBt, DIC, 40 °C, 1 h; (3) fatty acid, HOBt, HBTU, DIEA, 40 °C, 1.5 h; l. (1) 92.5% TFA, 2.5% DODT, 2.5% TIS, 2.5% H_2_O, 40 °C, 2 h.

#### Discovery of a Potent PTPN1/N2 Dual Inhibitor **Y5** Derived from BimBH3 Peptide

##### Evaluation of PTPN1/N2 Inhibitory Activity

Given the synergistic roles of PTPN1 and PTPN2 in insulin and leptin signaling pathway dephosphorylation, we investigated the dual-target inhibitory potential of the eight analogues. We assessed the inhibitory potency of DNP conjugated fatty acid analogues against human recombinant PTPN1/N2 through a two-stage screening process. Initial compound evaluation at 40 μM revealed significant inhibitors (>50% inhibition), which subsequently underwent IC_50_ determination (Table 1).

**Table 1:** PTPN1/2 Inhibitory Activity of the BimBH3 Peptide Analogues

	Conjugate group		Inhibitory activity (IC_50_, μM)	
Analogue	N-terminal	Raa^2^ substituent	PTPN1	PTPN2
**SM6**	C16 acid	Ala	0.960	nt^a^
**Y1**	C16 acid	Lys-DNP	0.629	nt
**Y2**	C16 acid	Dap-DNP	0.289	nt
**Y3**	C16 acid	Dab-DNP	0.265	nt
**Y4**	C16 acid	Orn-DNP	0.144	nt
**Y5**	C18 diacid	Lys-DNP	0.116	5.613
**Y6**	C18 diacid	Dap-DNP	0.483	nt
**Y7**	C18 diacid	Dab-DNP	0.438	nt
**Y8**	C18 diacid	Orn-DNP	0.200	nt

a nt: the IC_50_ values were not tested when inhibitory rates were <50% at a concentration of 40 μmol/L.

The DNP-modified mimetics all demonstrated markedly enhanced enzymatic inhibition compared to the lead compound **BimBH3-SM6**. All synthesized analogues showed improved PTPN1 inhibitory activity compared to the lead compound. In the C16 acid series (Y1–Y4), all compounds exhibited good PTPN1 inhibition (IC_50_ = 0.629–0.144 μM), but none showed detectable PTPN2 inhibition. In the present C18 diacid series, only **Y5** (bearing the longest Lys-DNP linker) displayed measurable PTPN2 inhibition. Notably, our previous work demonstrated that PTPN2 activity can also be achieved with certain C16-containing analogues when combined with an additional fatty-acid modification at Lys^2^ (e.g., **D5** in Zhang et al.).^46^ This suggests that PTPN2 engagement is determined by the combined effects of fatty-acid type, linker length, and the nature of the Lys^2^ substituent, rather than by any single structural feature.

**Y5** exhibits potent PTPN1 inhibition and moderate PTPN2 inhibition (∼48-fold selective). **Y5** inhibited PTPN1 with an IC_50_ of 116 nM, representing an approximately 8-fold increase in potency compared with the parent BimBH3 analogue. Importantly, **Y5** concurrently inhibited the closely related phosphatase PTPN2 with micromolar potency, establishing a dual-target inhibitory profile consistent with the coordinated regulation of insulin and leptin signaling by these enzymes. This enhanced activity likely originates from two structural advantages: (i) the octadecanedioic acid moiety promotes optimal target engagement through improved albumin-mediated binding, and (ii) the strategically placed DNP on the lysine side chain minimizes steric hindrance while preserving hapten accessibility.

##### Evaluation of Albumin Binding Capacity for BimBH3 Mimetic Peptides

To assess whether the fatty-acid modification conferred functional albumin binding, we evaluated the inhibitory activity of **Y5** in the presence of human serum albumin. Inclusion of 2% albumin resulted in a reduced PTPN1 inhibition activity, consistent with strong, reversible sequestration by albumin (Figure 2). This ∼50-fold decrease in potency is consistent with strong, noncovalent binding to the fatty acid-binding sites on albumin, a well-established mechanism for prolonging the circulatory half-life of peptide therapeutics, which could validate the pharmacokinetic rationale behind the fatty acid and DNP dual conjugation. This behavior is characteristic of long-acting peptide therapeutics and supports the premise that albumin binding contributes to sustained systemic exposure rather than diminishing intrinsic target engagement.

**Figure 2:**
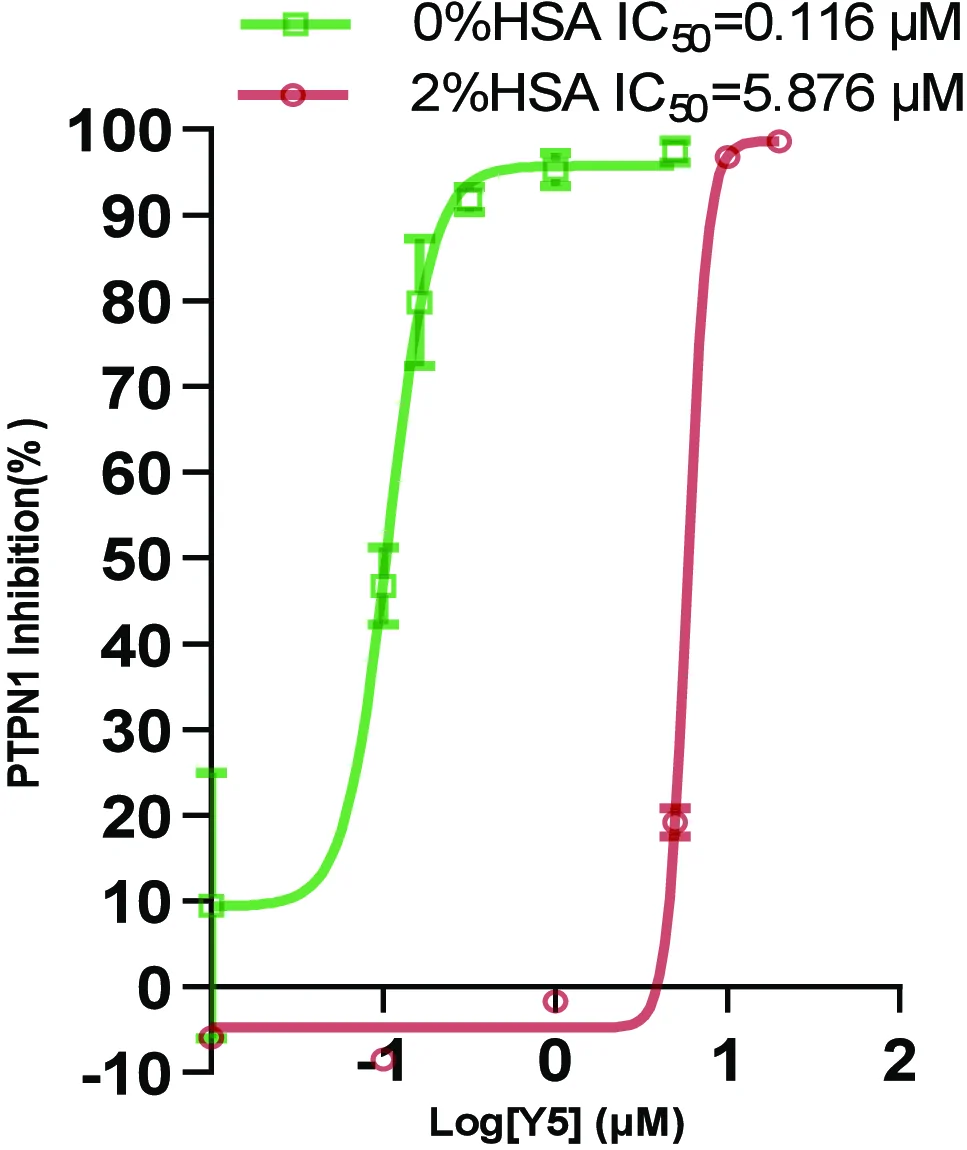
Albumin binding potential indicated by the *in vitro* inhibitory potency decrease of BimBH3 analogue **Y5** with 2% human serum albumin. The results are presented as the mean ± SD of 3 replicates.

### Structural Analysis of BimBH3 Analogues by Circular Dichroism

Circular dichroism (CD) spectroscopy was employed to characterize the secondary structure of BimBH3 mimetic peptides in the far-ultraviolet (UV) region (180–260 nm), a critical wavelength range for probing peptide conformational features. This technique enables rapid assessment of secondary structure composition and identification of functional conformations such as α-helices or β-sheets. Analysis of hapten-conjugated analogues Y1–Y8 in peptide-compatible buffer revealed minimal canonical secondary structure formation. CD spectra demonstrated low α-helical content across all analogues, with no observable signatures of ordered β-sheet or turn motifs. The structural profiles resembled those of small molecules, exhibiting predominantly disordered conformations (Figure 3).

**Figure 3:**
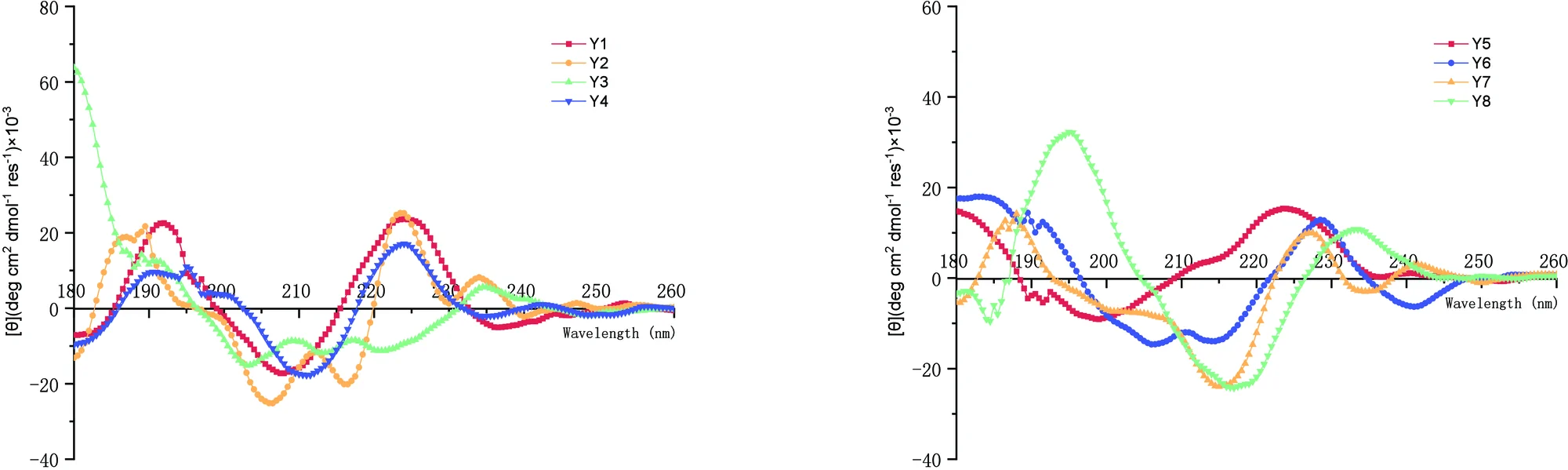
Far-UV CD spectra of BimBH3 analogues in aqueous buffer (0.5 mg/mL in 50 mM Tris-HCl buffer containing 100 mM NaCl, pH 7.4, 25 °C).

#### Molecular Docking Predicts the Mode of Action between **Y5** and PTPN1/N2

Molecular docking analyses further supported the dual inhibitory activity of **Y5** by revealing stable binding modes and large binding areas with the catalytic pockets of both PTPN1 and PTPN2 (Figure 4). **Y5** engaged extensive hydrogen-bonding networks and hydrophobic interactions with conserved residues critical for phosphatase activity, yielding predicted binding energies consistent with the observed biochemical inhibition. The ability of a single peptide scaffold to accommodate subtle structural differences between PTPN1 and PTPN2 provides a structural rationale for its concurrent targeting of both enzymes. In this study, we simulated the interaction patterns between PTPN1/2 and the BimBH3 Analogue **Y5**. The crystal structures of PTPN1 and PTPN2 were obtained from the Protein Data Bank (PDB IDs: 9CYO and 7UAD, with resolutions of 1.94 Å and 2.04 Å, respectively). The protein structures were prepared by removing all heteroatoms and crystallographic water molecules. Protonation states of ionizable residues were assigned at pH 7.4. A short energy minimization was then performed primarily to alleviate any atomic clashes introduced during the addition of hydrogen atoms. The 3D structure of the BimBH3 analogue **Y5** was sketched in ChemDraw 2023 and subsequently geometry-optimized using the MMFF94 force field in Chem3D to obtain a low-energy conformation. Its predominant ionization state at pH 7.4 was assigned to ensure physiological relevance. Molecular docking was performed using AutoDock Vina, and the results are depicted in Figure 4.

**Figure 4:**
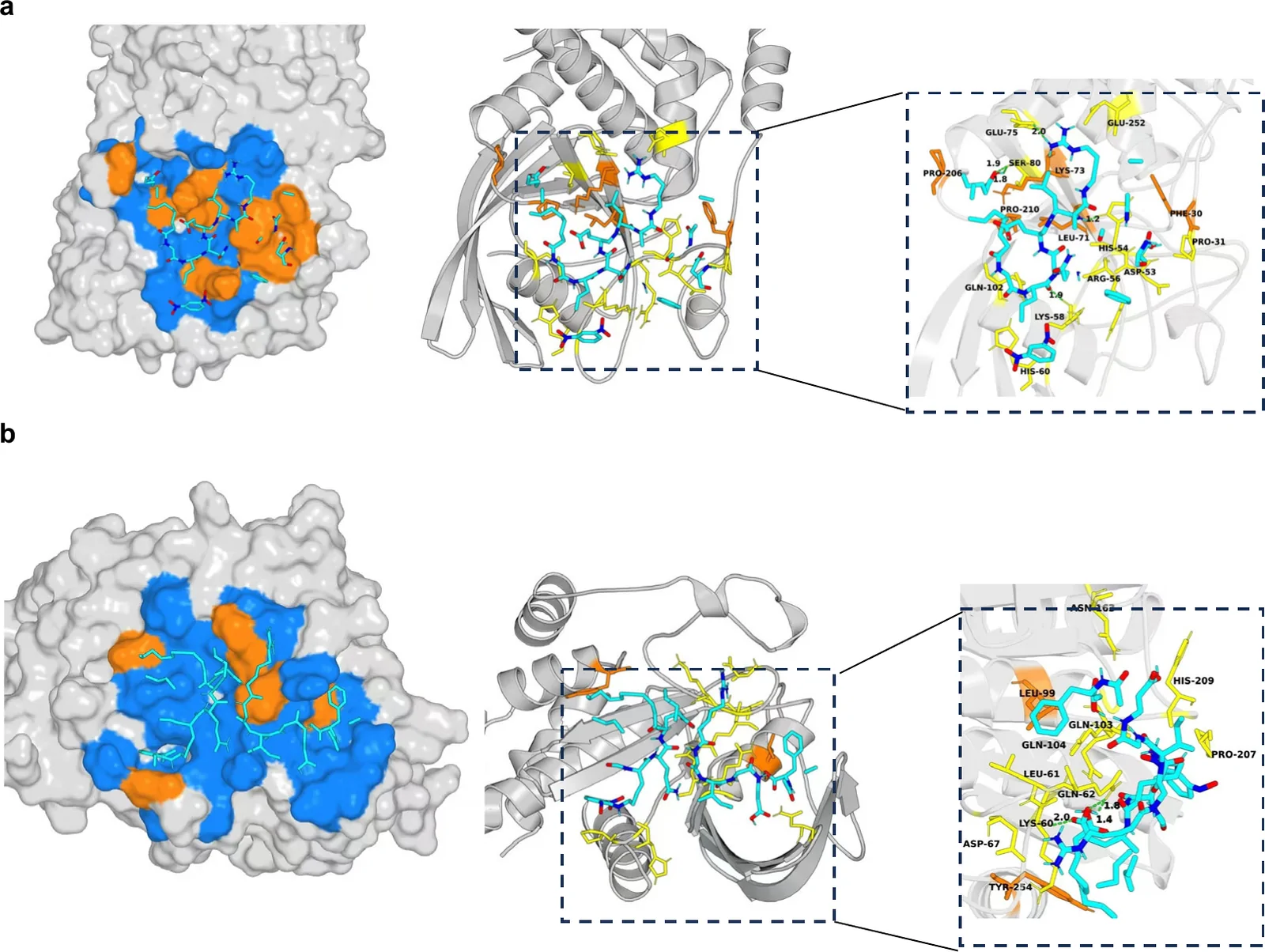
Molecular stimulation and docking illustrations of interactions between **Y5** and (a) PTPN1 and (b) PTPN2.

The docking results revealed binding free energies of −5.8 kcal/mol and −5.2 kcal/mol for **Y5** with PTPN1 and PTPN2, respectively, indicating relatively favorable binding affinity consistent with *in vitro* inhibition for both targets. These computational results are consistent with the *in vitro* enzyme inhibition assays, further supporting the potent inhibitory potential of **Y5** against PTPN1/2. Specifically, the high-affinity binding of **Y5** to the active sites of PTPN1 and PTPN2 is stabilized by synergistic combinations of extensive hydrogen bonds and hydrophobic interactions. For PTPN1, a dense network of hydrogen bonds is formed with key residues, including P31, D53, H54, R56, K58, H60, E75, S80, K102, and K252 (the yellow part in the Figure 4a). This intricate hydrogen-bonding network is critical for specifically anchoring and stabilizing the compound within the binding pocket. Concurrently, hydrophobic interactions involving residues such as F30, P31, D53, H54, K58, L71, K73, P206, and P210 further stabilize the complex by encapsulating the nonpolar regions of **Y5** (the orange part in the Figure 4a). Notably, several critical residues, such as H54 and K58, play dual roles: their polar atoms form hydrogen bonds, while their aliphatic/aromatic moieties contribute to hydrophobic effects. Similarly, for PTPN2, an even more extensive network of hydrogen bonds is formed with residues including K60, 61L, Q62, D67, Q103, Q104, N163, P207, and H209 (the yellow part in the Figure 4b). This is complemented by a hydrophobic shell formed by residues such as K60, Q62, L99, P207, and Y254, which interact with the nonpolar regions of **Y5** (the orange part in the Figure 4b).

#### Molecular Dynamics Simulations Reveal Stable Binding of **Y5** to PTPN1/N2

To further validate the binding modes predicted by molecular docking and to assess the stability and specificity of **Y5** interactions with PTPN1 and PTPN2 from a dynamic perspective, we performed 50 ns all-atom molecular dynamics simulations. The following results systematically compare the binding behavior of **Y5** with the two phosphatases across multiple metrics, including center-of-mass distance, minimum atomic distance, ligand and protein backbone root-mean-square deviation (RMSD), and energy fluctuations (Figure 5).

**Figure 5:**
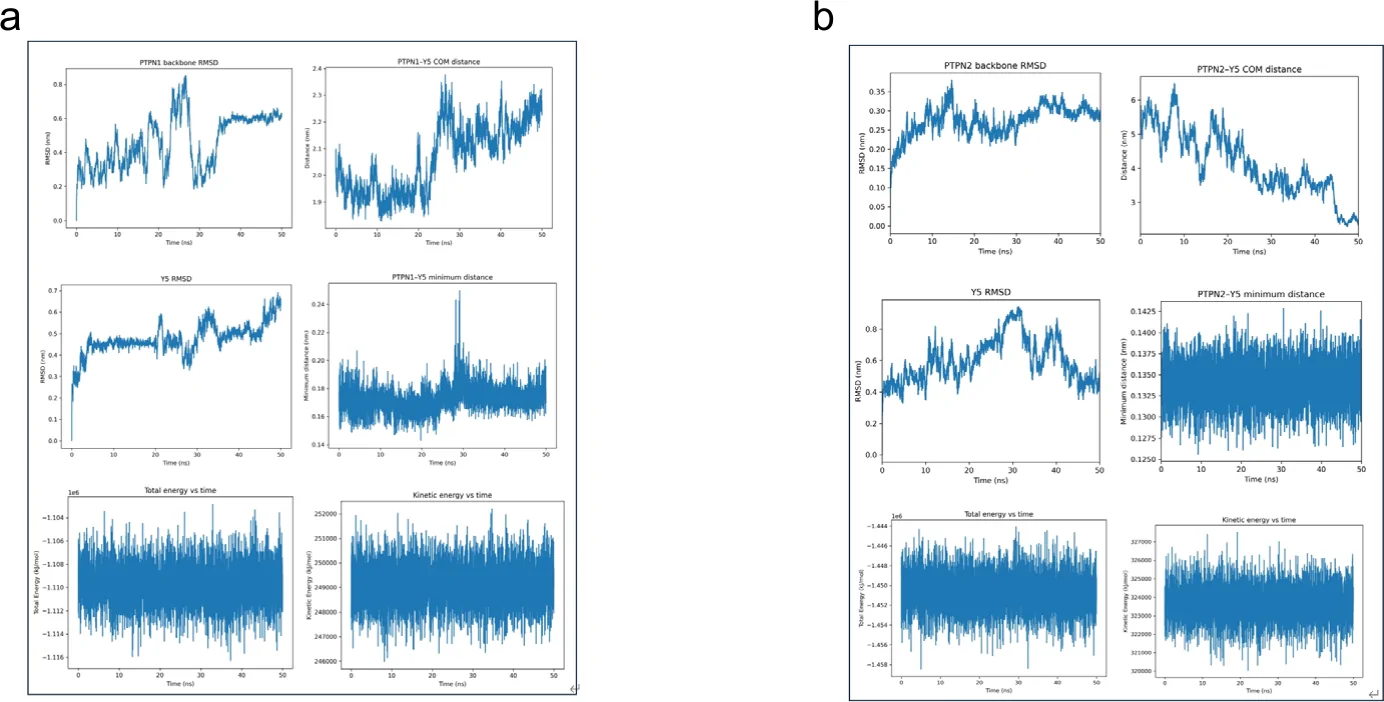
Molecular dynamics simulation analysis of **Y5** in complex with (a) PTPN1 and (b) PTPN2. Plots show the time evolution of center-of-mass distance, minimum atomic distance, ligand RMSD, protein backbone RMSD, total energy, and kinetic energy.

For the PTPN1–**Y5** complex, the center-of-mass (COM) distance remained relatively stable within the range of 1.9–2.3 nm, predominantly fluctuating between 2.0 and 2.3 nm, with no sustained increase or directional drift. This indicates that **Y5** remains stably associated with PTPN1 without undergoing dissociation. Consistently, the minimum atomic distance was maintained between 0.145 and 0.200 nm (primarily 0.15–0.18 nm), which is well below the typical van der Waals contact distance (∼0.3–0.4 nm), demonstrating persistent close contacts likely mediated by hydrogen bonding and hydrophobic interactions. These observations are in agreement with the molecular docking results. From a structural perspective, the backbone RMSD of PTPN1 fluctuated within 0.2–0.8 nm without any continuous upward trend, suggesting that the overall protein structure remained stable without significant unfolding or large-scale rearrangements. The observed fluctuations likely reflect inherent conformational flexibility, potentially associated with an induced-fit binding mechanism. Meanwhile, the RMSD of **Y5** increased rapidly during the initial phase and subsequently stabilized within the range of 0.4–0.6 nm, indicating that the ligand underwent conformational adaptation before adopting a relatively stable binding conformation. In terms of system stability, the kinetic energy remained steady at approximately 2.49 × 10^5^ kJ/mol, while the total energy fluctuated minimally around −1.11 × 10^6^ kJ/mol, indicating that the system had reached thermodynamic equilibrium. Collectively, these results demonstrate that **Y5** forms a stable and tightly bound noncovalent complex with PTPN1.

In contrast, for the PTPN2–**Y5** complex, the center-of-mass (COM) distance fluctuated between approximately 3.0 and 6.0 nm and exhibited a decreasing trend from an initially larger separation, indicating that the ligand gradually approached the protein and established binding during the simulation. Meanwhile, the minimum atomic distance remained consistently within 0.125–0.142 nm, demonstrating persistent and close atomic contacts between the two, which provide direct evidence for stable noncovalent interactions. Furthermore, the RMSD of **Y5** fluctuated within the range of 0.35–0.85 nm, suggesting that the ligand underwent conformational adjustments during the binding process. In comparison, the backbone RMSD of PTPN2 increased rapidly at the initial stage and stabilized after approximately 10 ns, maintaining fluctuations within 0.25–0.35 nm. This indicates that the protein structure remained largely stable without significant perturbations throughout the simulation. In terms of energetics, both the kinetic energy and total energy exhibited stable fluctuations. The kinetic energy remained within 320,000–327,000 kJ/mol, while the total energy fluctuated around −1.45 × 10^6^ kJ/mol without any noticeable drift, suggesting that the system had reached a well-equilibrated thermodynamic state. Overall, these results indicate that **Y5** forms a stable interaction complex with PTPN2 during the simulation.

#### **Y5** *In Vitro* Proteolytic Stability against DPP-IV and Plasma

Dipeptidyl peptidase-4 (DPP-IV), a serine protease responsible for rapid peptide degradation, exhibits substrate specificity for polypeptides containing proline (Pro) or alanine (Ala) at the penultimate N-terminal position. Its catalytic triad (Ser630, Asp708, His740) and hydrophobic substrate-binding pocket enable precise recognition of conformations matching X-Pro/Ala motifs. Mechanistically, DPP-IV cleaves the peptide bond between residues 2 and 3 of susceptible polypeptides, releasing N-terminal dipeptides (e.g., X-Pro or X-Ala fragments). Structural analysis of the native BimBH3 peptide revealed an Ala residue at position 2, rendering it vulnerable to DPP-IV-mediated proteolysis. To identify this degradation pathway and improve metabolic stability, we strategically substituted this Ala residue with a noncanonical amino acid designed to disrupt DPP-IV recognition while preserving target engagement.

To assess the resistance of mimetic peptide **Y5** to DPP-IV-mediated degradation, we conducted *in vitro* degradation resistance assays. As shown in Figure 6a, **Y5** exhibited significantly improved stability with a half-life of 13.78 h, representing a 45-fold increase over the original **BimBH3** peptide and a 4.5-fold enhancement compared to **SM-6**. These results demonstrate that the dual strategy of targeted amino acid substitution and DNP conjugation effectively enhances proteolytic stability by mitigating DPP-IV recognition and cleavage.

**Figure 6:**
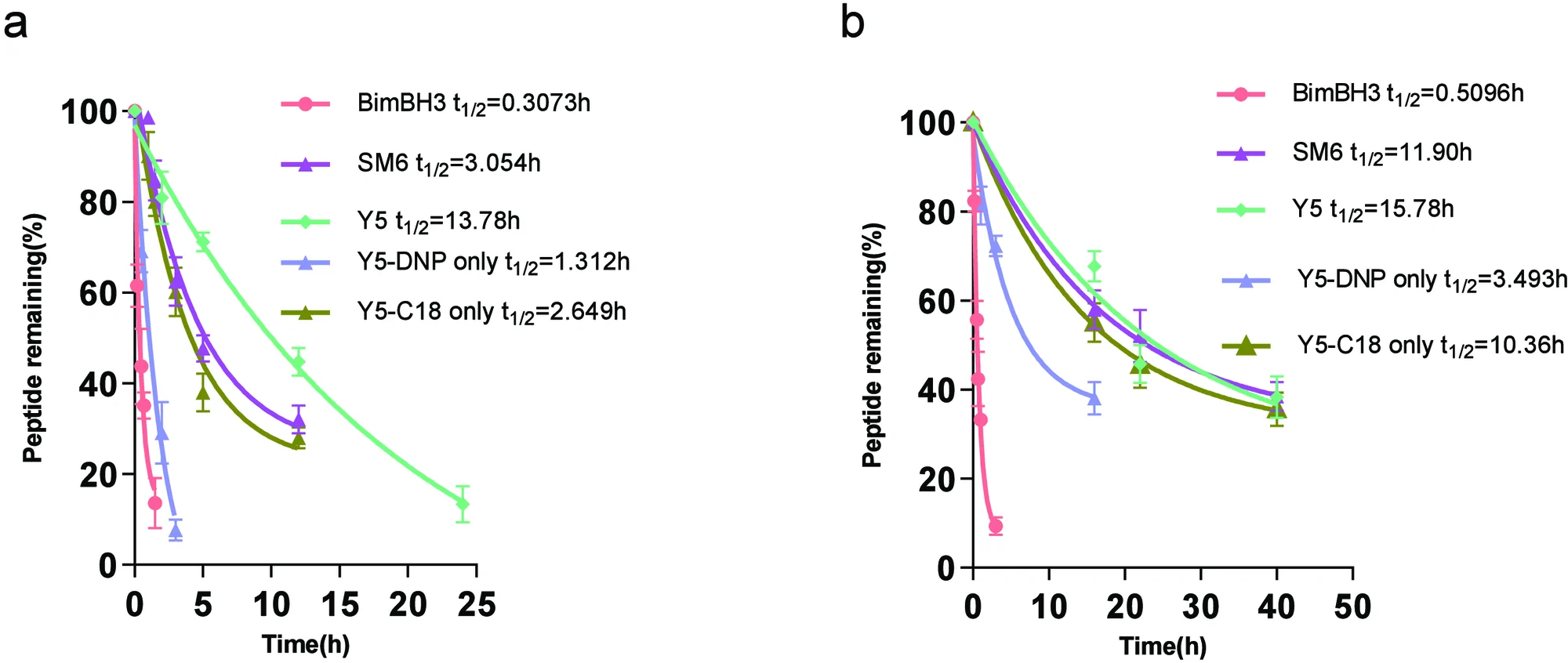
*In vitro* biostability of BimBH3 mimetic peptides against (a) DPP-IV degradation and (b) plasma. The results shown are mean ± SD (*n* = 3).

Prolonging drug half-life enhances therapeutic outcomes by improving patient compliance, stabilizing systemic exposure, and sustaining efficacy-critical advantages for chronic disease management. Based on **Y5**’s demonstrated resistance to DPP-IV degradation (*in vitro* t_1/2_ = 13.78 h), we further evaluated its pharmacokinetic stability in rat plasma (Figure 6b). **Y5** exhibited markedly improved plasma stability with a half-life of 15.78 h, representing a 31-fold increase over **BimBH3** and a 1.33-fold improvement compared to **SM-6**.

To further clarify the respective contributions of the fatty acid and DNP modifications to stability, we synthesized two control analogues: **Y5**-DNP only (containing only the Lys^2^-DNP modification, with no fatty acid) and **Y5**-C18 only (containing only the N-terminal C18 diacid modification, with Ala^2^ and no DNP). Their DPP-IV stability and mouse plasma stability were then evaluated under identical conditions. For DPP-IV stability, the half-lives of the parent BimBH3, SM-6, **Y5**, **Y5**-DNP only, and **Y5**-C18 only were 0.307, 3.054, 13.78, 1.312, and 2.649 h, respectively (Figure 6a). For mouse plasma stability, the half-lives of the same compounds were 0.510, 11.90, 15.78, 3.493, and 10.36 h, respectively (Figure 6b). The dual-modified **Y5** exhibited substantially longer half-lives than either single-modified control. The fatty-acid-only analogue (**Y5**-C18 only) already provided considerable stabilization, and the DNP-only analogue (**Y5**-DNP only) also afforded a modest improvement. Importantly, the combination of the two modifications achieved the greatest effect, demonstrating a synergistic contribution of the fatty acid and DNP modifications to overall stability. In summary, **Y5** was a promising long-acting therapeutic candidate for T2DM and/or obesity therapy, with potential of cellular uptake and function, and maintenance of stable drug levels and minimize dosing frequency in clinical applications.

#### **Y5** Restores Cellular Insulin and Leptin Signaling by Enhancing Phosphorylation of Downstream Signaling Molecules

Insulin and leptin resistance represent convergent signaling defects underlying hyperglycaemia and excess adiposity. At the molecular level, impaired phosphorylation of insulin receptor substrates and downstream AKT, together with blunted JAK2-STAT3 activation, are hallmarks of disrupted insulin and leptin responsiveness. Because PTPN1 and PTPN2 directly dephosphorylate key nodes within both pathways, we hypothesized that effective dual inhibition would restore signaling competence under insulin- and leptin-resistant conditions.

A critical step in this process is the phosphorylation of insulin receptor substrate 1 (IRS-1) and its key downstream signaling molecules, such as AKT. Phosphorylated AKT (p-AKT) subsequently serves as the primary mediator of glucose uptake and metabolic regulation. Similarly, the leptin signaling pathway is also important for glucose metabolism. Impaired transduction of the JAK2-STAT3 pathway downstream of leptin signaling is a central feature of leptin resistance, specifically manifested as a significant reduction in tyrosine phosphorylation levels of STAT3. Therefore, detecting the level of p-STAT3 is a key indicator for assessing leptin sensitivity in cells. A lower levels of p-IRS1, p-AKT and p-STAT3 indicate impaired insulin/leptin signaling. Consequently, increased levels of p-IRS1, p-AKT, and p-STAT3 can signify restored insulin/leptin sensitivity. PTPN1 negatively regulates insulin signal transduction by dephosphorylating IRS-1 and exerts a negative regulatory effect on the leptin signaling pathway by dephosphorylating JAK2 in the JAK2-STAT3 pathway.

We sought to determine whether the analogue **Y5** enhances the phosphorylation of downstream signal molecules via PTPN1 inhibition to restore signaling sensitivity in the insulin and leptin pathways.^19^ As shown in Figure 7a, the results indicated that treatment with **Y5** resulted in a robust and dose-dependent restoration of insulin and leptin signaling. Specifically, **Y5** markedly increased the phosphorylation of IRS-1 and AKT in response to insulin stimulation, while simultaneously enhancing STAT3 phosphorylation following leptin exposure. These effects were observed across a broad concentration range and occurred without detectable changes in total PTPN1 protein levels, indicating that **Y5** acts by functionally relieving phosphatase-mediated signal attenuation rather than altering phosphatase expression.

**Figure 7:**
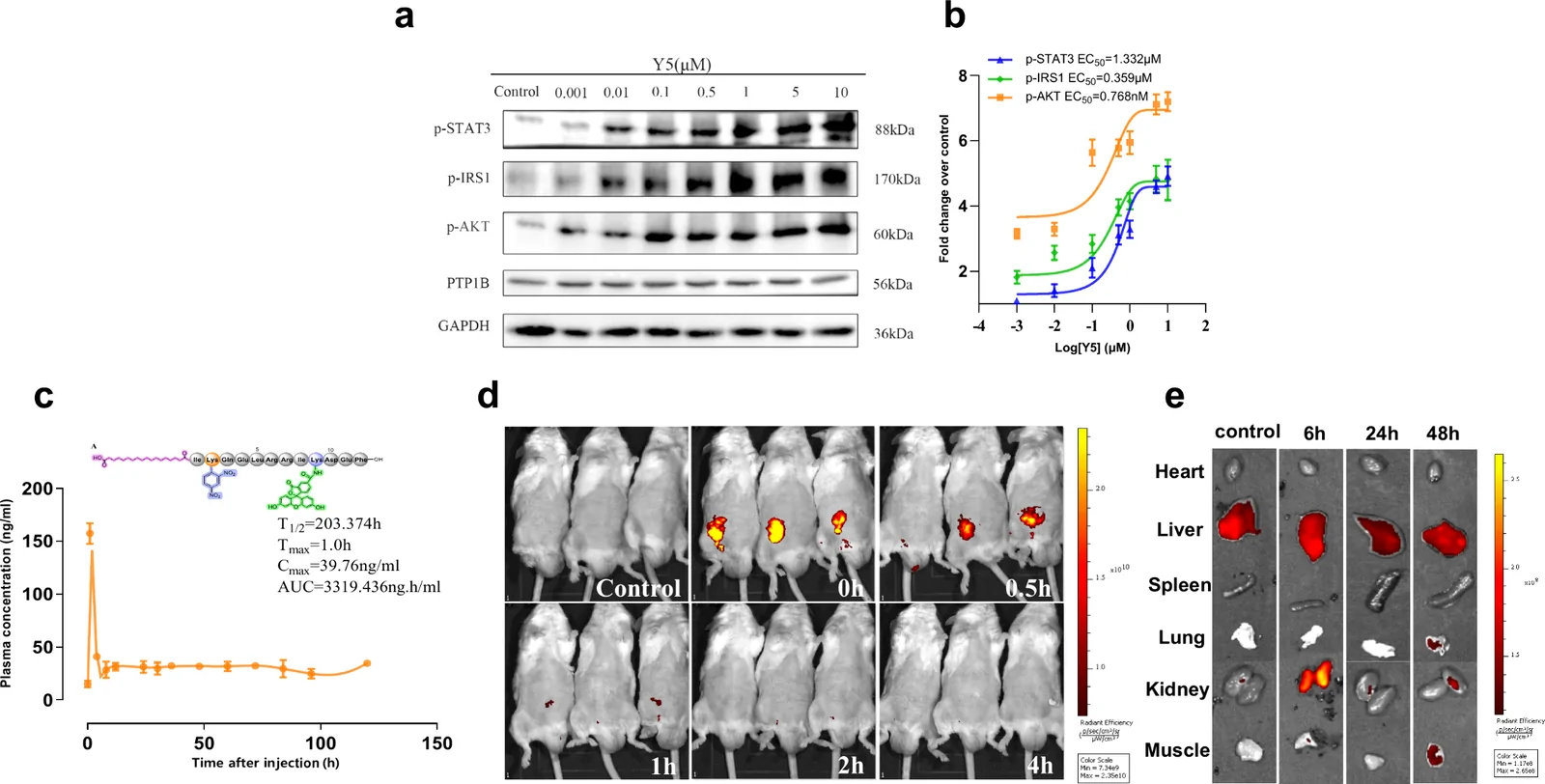
(a,b) Western blot analysis of p-STAT3, p-AKT, and p-IRS1 levels in HepG2 cells treated with BimBH3 analogue **Y5** at 0.001, 0.01, 0.1, 0.5, 1, 5, and 10 μM for 24 h. The control group exhibits insulin resistance induced by palmitic acid with equivalent volume of DMSO, and GAPDH was used as a loading control to ensure equal protein loading across samples. The changes in the levels of p-IRS1, p-AKT, and p-STAT3 in HepG2 cells treated with different concentrations of BimBH3. The results shown are mean ± SD (*n* = 3). (c) Structure of the fluorescein (Flu)-labeled analogue **Lys** ^**9**^ **-Flu-Y5** incorporating 5-carboxyfluorescein (5-FAM), and *in vivo* pharmacokinetic investigation of the Flu-labeled analogue **Lys** ^**9**^ **-Flu-Y5** in SD rats following a single subcutaneous dose of 100 nmol/kg. Blood samples were collected at various time points for fluorescence quantification. Data are depicted as the mean ± SEM (*n* = 3). (d,e) *In vivo* and *ex vivo* distributions profiles of **Lys** ^**9**^ **-Flu-Y5** at different time points (0, 0.5, 1, 2, and 4 h) post subcutaneous injection determined using the optical *in vivo* imaging system. The mice were administered **Lys** ^**9**^ **-Flu-Y5** at a dose of 100 nmol/kg.

Quantitative analysis revealed that **Y5** restored downstream signaling with high potency, yielding half-maximal effective concentrations in the nanomolar to low micromolar range for AKT, IRS-1 and STAT3 phosphorylation. The coordinated enhancement of insulin- and leptin-responsive signaling nodes supports a model in which dual PTPN1/PTPN2 inhibition reinstates hormonal sensitivity at the cellular level. Notably, the simultaneous restoration of insulin and leptin signaling by **Y5** distinguishes this mechanism from incretin-based therapies, which primarily act through appetite suppression and central satiety pathways. By directly enhancing intracellular hormone sensitivity, **Y5** provides a promising mechanistic framework capable of exhibiting glycemic control and body-weight reduction observed *in vivo*, independent of changes in food intake.

#### **Y5** Pharmacokinetic Profiles and Biodistribution in Mice

Restoration of insulin and leptin signaling represents a necessary but not sufficient condition for achieving durable metabolic benefit *in vivo*. Because both pathways are dynamically regulated and subject to rapid dephosphorylation, sustained target engagement is essential for translating intracellular signaling correction into long-term glycemic control and weight reduction. We therefore investigated whether **Y5** possesses the pharmacokinetic properties required to support prolonged inhibition of PTPN1 and PTPN2 *in vivo*.

To evaluate the pharmacokinetic properties of fatty acid- and DNP-conjugated BimBH3 analogues, we conducted preliminary studies using the fluorescently labeled analogue **Lys** ^**9**^ **-Flu-Y5 (** Figure 7c) following previously established methods.^44–46^ Plasma concentration–time profiles were analyzed via noncompartmental modeling to determine pharmacokinetic parameters (Figure 7c). Following subcutaneous administration (100 nmol/kg), **Lys** ^**9**^ **-Flu-Y5** exhibited an exceptionally long half-life (T_1/2_ = 203.4 h) and a substantial total systemic exposure (AUC = 3319.436 ng·h/mL). These two key characteristics collectively support its strong potential as long-acting PTPN1/N2 inhibitor for glycemic control. **Lys** ^**9**^ **-Flu-Y5** also demonstrated a remarkably short time to reach peak concentration (T_max_ = 1.0 h), indicating rapid entry into the systemic circulation and prompt onset of action. Furthermore, its lower maximum plasma concentration (C_max_ = 39.76 ng/mL) suggests a potentially wider therapeutic window and improved safety profile, as lower peak exposures are typically associated with a reduced risk of acute toxicities. These favorable pharmacokinetic properties indicated that **Y5** could achieve long-acting (e.g., once-weekly or even once-monthly) treatment regimen for type 2 diabetes mellitus and obesity. The prolonged pharmacokinetic profile likely arises from the following possible synergistic mechanisms: (1) fatty acid-mediated albumin binding reducing renal clearance, (2) enhanced resistance to DPP-IV degradation, and (3) DNP conjugation improving metabolic stability through structural shielding.

To evaluate the *in vivo* absorption, distribution, and retention of fatty acid and DNP dual modified BimBH3 analogues, we also performed real-time biodistribution analysis using fluorescently labeled **Lys** ^**9**^ **-Flu-Y5**. Noninvasive fluorescence imaging revealed rapid subcutaneous absorption, with pronounced signals at the injection site at 0 and 0.5 h postadministration (Figure 7d). The marked reduction in fluorescence intensity at the injection site by 0.5 h correlated with systemic distribution rather than degradation, consistent with the pharmacokinetic profile observed in Figure 7f. Longitudinal tissue imaging demonstrated time-dependent biodistribution patterns over 6–48 h. **Lys** ^**9**^ **-Flu-Y5** exhibited preferential accumulation in metabolic and excretory organs, with highest fluorescence intensities observed in the liver, kidneys, and skeletal muscle. This organ-specific distribution aligns with the dual-modification strategy for long-acting peptide drugs,^47^ where albumin binding likely mediates prolonged tissue retention while maintaining metabolic elimination pathways. The predominant hepatic and muscular accumulation of **Lys** ^**9**^ **-Flu-Y5** aligns with the tissue-specific expression profiles of its molecular targets PTPN1 and PTPN2, protein tyrosine phosphatases with elevated activity in liver and skeletal muscle. The combination of sustained systemic exposure and appropriate tissue distribution provides a mechanistic basis for durable modulation of insulin and leptin signaling *in vivo*.

Together, these stability and pharmacokinetic properties establish **Y5** as a long-acting dual PTPN1/PTPN2 inhibitor capable of maintaining prolonged intracellular target inhibition. This temporal dimension is critical for translating acute signaling restoration into sustained improvements in glycemic control and body-weight regulation, providing a necessary foundation for evaluating the *in vivo* metabolic efficacy of **Y5**.

#### Glycemic Control Efficacy and Weight-Gain Reduction Benefit of **Y5** in db/db Mice

The restoration of insulin and leptin signaling at the cellular level raises a critical translational question whether sustained dual inhibition of PTPN1 and PTPN2 could produce durable metabolic benefits *in vivo* under conditions of severe insulin resistance. To address this, we evaluated the long-term efficacy of **Y5** in leptin receptor-deficient db/db mice, a well-established model of advanced type 2 diabetes characterized by hyperglycaemia, hyperinsulinaemia, and progressive weight gain. Based on promising results from preliminary experiments, the long-term antidiabetic and weight-loss benefit of the candidate analogue **Y5** was further evaluated in a 7-week study using db/db diabetic mouse models (Figure 8a). Mice received once-weekly subcutaneous injections of **Y5** at doses of 50 nmol/kg (0.10 mg/kg), 100 nmol/kg (0.20 mg/kg), and 200 nmol/kg (0.40 mg/kg). Semaglutide (100 nmol/kg, MW = 4113.58, i.e., 0.41 mg/kg) was used as the positive control. Fasting blood glucose monitoring indicated that at most time points, once-weekly s.c. administration of **Y5** at all three dose levels showed obvious glycemic control efficacy, with trends consistent with that observed in the semaglutide treatment group (Figure 8b). This superior performance was reflected in the sustained suppress of gain of fasting blood glucose levels observed over the long-term treatment period, suggesting that **Y5** could offer a competitive alternative therapy with enhanced therapeutic benefits in long-term diabetes management.

**Figure 8:**
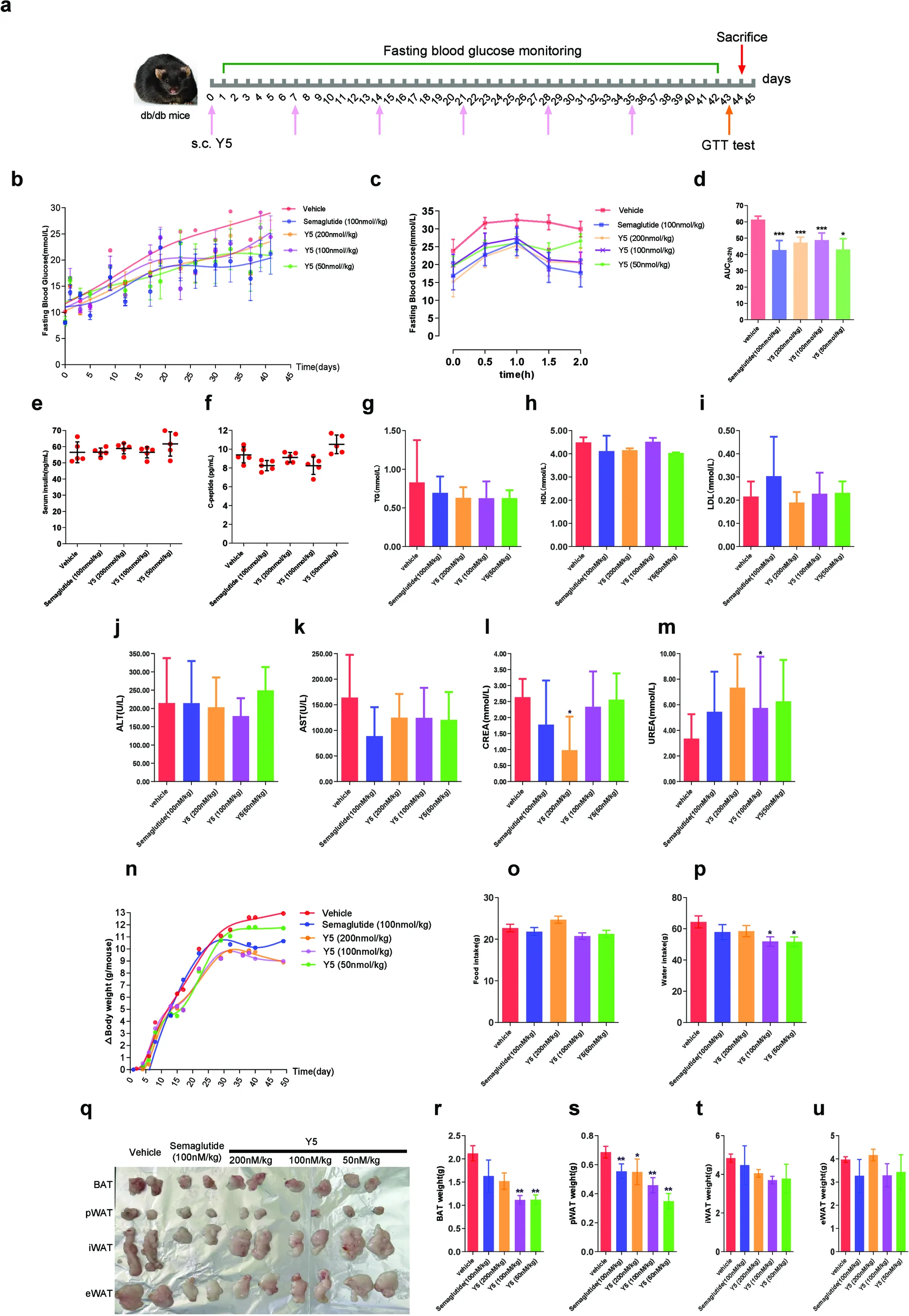
**Y5** improves glucose homeostasis and metabolic parameters in db/db mice. (a) Study design and dosing scheme. (b) Fasting blood glucose levels measured in db/db mice receiving once-weekly subcutaneous injections of saline, semaglutide (100 nmol/kg), or **Y5** (50, 100, or 200 nmol/kg) over a 7-week treatment period. (c) Oral glucose tolerance test (OGTT) glucose excursion curves following 7 weeks of treatment. (d) Corresponding area under the curve (AUC) analysis of OGTT. (e,f) Serum insulin (e) and C-peptide (f) levels measured at the end of the study, indicating improved insulin sensitivity in **Y5**-treated mice. (g–i) Plasma lipid profiles following 7 weeks of treatment, including triglycerides (TG) (g), high-density lipoprotein cholesterol (HDL-C) (h), and low-density lipoprotein cholesterol (LDL-C) (i). (j–m) Safety assessment based on serum biochemical parameters measured at study termination. Liver function markers include alanine aminotransferase (ALT) (j) and aspartate aminotransferase (AST) (k); renal function markers include creatinine (CREA) (l) and urea (UREA) (m). (n) Body-weight progression during the treatment period. (o,p) Food and water intake. (q) Representative images of dissected adipose tissues, including brown adipose tissue (BAT), perirenal white adipose tissue (pWAT), inguinal white adipose tissue (iWAT), and epididymal white adipose tissue (eWAT). (r–u) Quantification of adipose tissue weights across treatment groups. All data are presented as the mean ± SD, *n* = 5 mice per group. *p < 0.05, **p < 0.01, ***p < 0.001 versus vehicle control.

Oral glucose tolerance testing (OGTT) revealed marked reductions in glucose excursion and area under the curve compared with vehicle-treated controls (Figure 8c and 8d), indicating that **Y5** improves the capacity to handle acute glycemic challenges. To further assess the mechanism underlying glycemic improvement, we examined circulating insulin and C-peptide levels at the end of the treatment period. **Y5**-treated mice exhibited significantly reduced serum insulin and C-peptide concentrations relative to controls (Figure 8e and 8f), consistent with enhanced insulin sensitivity rather than increased insulin secretion. This profile contrasts with insulinotropic or incretin-based mechanisms and aligns with the restoration of intracellular insulin signaling observed *in vitro*.

Given the downward trend in body weight in the **Y5** treatment groups, lipid metabolism parameters were also measured after long-term treatment. Results in Figure 8g–8i show that plasma levels of low-density lipoprotein cholesterol (LDL-C), triglycerides (TG), and high-density lipoprotein cholesterol (HDL-C) in the semaglutide and **Y5** treatment groups were slightly lower than those in the saline control group. These results suggest that **Y5** can improve lipid metabolism in db/db mice to some extent. Furthermore, relevant safety indicators were assessed, including hepatotoxicity markers (AST, ALT) and nephrotoxicity markers (CREA, UREA) (Figure 8j–8m). It is worth noting that the CREA level in the 0.5 μmol/kg **Y5** group was significantly lower than that in the control group (*p* < 0.05), while the UREA level was higher. Collectively, these data suggest that **Y5** was well tolerated and did not exhibit significant toxicity during the long-term treatment period, supporting the feasibility of sustained PTPN1/PTPN2 inhibition *in vivo*. These observations indicate that the metabolic benefits of **Y5** are not accompanied by detectable organ toxicity under the dosing regimen employed.

In parallel with glycemic improvement, **Y5** treatment attenuated body-weight gain in db/db mice over the course of the study (Figure 8n) without reducing food intake (Figure 8o). Although the magnitude of weight reduction was modest relative to incretin-based therapy, **Y5** consistently slowed the progressive weight increase characteristic of this model, suggesting a direct metabolic effect rather than secondary weight loss driven by reduced caloric intake. To further investigate the effect of **Y5** on adipogenesis, mice with obvious weight-gain reduction were euthanized via CO_2_ inhalation at the end of the experiment, and various adipose tissues were isolated and examined. Analysis of adipose tissue depots (Figure 8q) revealed that **Y5** selectively reduced fat mass across multiple white adipose tissue compartments, including perirenal and inguinal depots, as well as brown adipose tissue (Figure 8q–8u). Specifically, after **Y5** treatment, the mass of brown adipose tissue (BAT), perirenal white adipose tissue (pWAT), inguinal white adipose tissue (iWAT), and epididymal white adipose tissue (eWAT) in obese mice showed a decreasing trend. Notably, the BAT and pWAT mass in mice treated with 100 nmol/kg and 50 nmol/kg **Y5** were significantly lower than those in the control group (*p* < 0.01, Figure 8r and 8s). These reductions indicate that **Y5**-mediated modulation of body weight is driven primarily by decreased adiposity rather than loss of lean mass, consistent with enhanced metabolic efficiency downstream of improved insulin and leptin signaling. Further investigations in long-term and chronic obesity models (e.g., diet-induced obesity mice) are imperative to validate the weight-loss therapeutic potential of **Y5**.

#### **Y5** Induces Weight Loss and Improves Metabolic Health in DIO Mice

Leptin resistance is a defining feature of common obesity, we next examined whether dual inhibition of PTPN1 and PTPN2 could overcome established metabolic dysfunction in diet-induced obese (DIO) mice. Unlike db/db mice, which lack functional leptin receptor signaling, DIO mice retain an intact but resistant leptin axis, thereby providing a stringent model to assess whether restoration of leptin sensitivity translates into meaningful weight loss independent of appetite suppression.

Mice received subcutaneous administration of **Y5** at doses of 25, 50, or 75 nmol/kg, or vehicle control, once every 3 days for a total treatment period of 6 weeks, followed by a 2-week treatment-free observation phase. Body weight and food intake were monitored throughout the study. Notably, **Y5**-induced weight loss was comparable in magnitude to that achieved with semaglutide, despite fundamentally distinct mechanisms of action. Weight reduction became evident within the first 2 weeks of treatment and was maintained throughout the dosing interval, consistent with long-acting target engagement. As illustrated in the Figure 9c and 9d, all **Y5**-treated groups exhibited significant weight loss compared to the vehicle control group. At the end of the 6-week treatment period, the high-dose group (75 nmol/kg) showed a reduction of −15.63%, while the medium- (50 nmol/kg) and low-dose (25 nmol/kg) groups exhibited reductions of −6.87% and −7.85%, respectively. Notably, this weight-lowering effect became even more pronounced during the 2-week off-treatment period, with the high-, medium-, and low-dose groups achieving weight reductions of −17.21%, −7.14%, and −7.92%, respectively, indicating a sustained and enhanced efficacy after drug withdrawal. Analysis of cumulative food intake revealed no significant differences between any **Y5**-treated groups and the vehicle control, suggesting that appetite suppression was not a contributing mechanism. In addition, serum insulin (Figure 9e) and leptin (Figure 9f) levels measured at the end of the study showed no significant improvement in insulin and leptin secretion in **Y5** treated DIO mice, indicating that the significant weight-loss efficacy was attributable to the amelioration of insulin and leptin resistance. These results were consistent with the cellular effects of **Y5** treatment, which significantly ameliorated insulin and leptin resistance.

**Figure 9:**
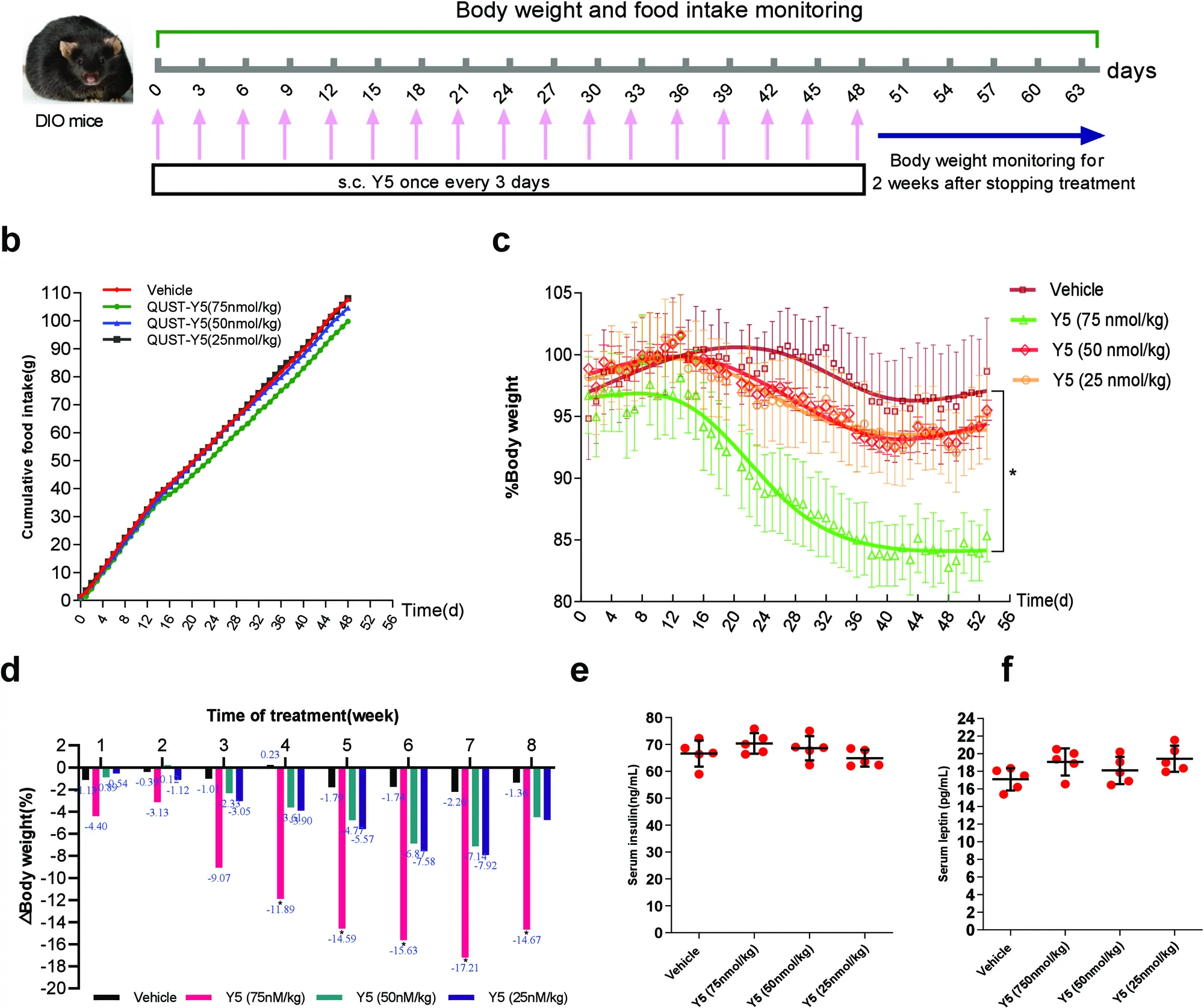
(a) Schematic representation of the experimental timeline in diet-induced obese (DIO) mice, comprising a 6-week treatment phase (**Y5** subcutaneous administration once every 3 days) followed by a 2-week off-treatment monitoring period. (b) Cumulative food intake measured across the 6-week treatment period. (c,d) Longitudinal percentage change in body weight over 8 weeks (c) and specific quantification of weight change at the conclusion of the 6-week treatment phase and the 2-week off-treatment phase (d). (e,f) Serum insulin (e) and leptin (f) levels measured at the end of the study showed no significant improvement in insulin and leptin secretion in **Y5**-treated DIO mice. Data are presented as the mean ± SD (*n* = 5 mice per group). *p < 0.05, **p < 0.01 vs vehicle.

As illustrated in Figure 9b, treatment with **Y5** did not significantly alter cumulative food consumption in DIO mice. Food intake remained comparable to vehicle-treated controls throughout the study, indicating that the observed weight loss might not be driven by reduced caloric intake. These findings demonstrate that **Y5** induces body-weight reduction through a potential appetite-independent mechanism as indicated by unchanged food intake.

Currently, GLP-1 receptor agonists, primarily function through potent and sustained suppression of central appetite, leading to reduced energy intake. This mechanism is frequently associated with gastrointestinal adverse effects, including nausea and vomiting, which can compromise patient adherence and quality of life. In comparison, **Y5** induces body-weight reduction through a mechanism that is not primarily driven by reduced caloric intake, as indicated by unchanged cumulative food intake; however, direct measurements of energy expenditure are needed to establish the underlying mechanism. This innovative mechanism, independent of chronic appetite suppression, may effectively circumvent the limitations associated with sustained satiety induction. The unique pharmacological profile of **Y5** underscores its considerable potential and distinctive therapeutic value in the development of next-generation antiobesity agents.

## Discussion

Current long-acting pharmacotherapies capable of treating both type 2 diabetes and obesity are dominated by incretin-based agents, which primarily achieve efficacy through sustained suppression of appetite. While clinically effective, this mechanism is intrinsically linked to gastrointestinal adverse effects, compromised eating-related quality of life, and a pronounced tendency toward metabolic rebound upon treatment discontinuation. In this study, we establish dual inhibition of PTPN1 and PTPN2 as a fundamentally distinct therapeutic strategy that dissociates weight loss and glycemic control from appetite suppression. By restoring intracellular insulin and leptin signaling, the long-acting peptide **Y5** achieves durable metabolic benefits in multiple mouse models of diabetes and obesity without reducing food intake, thereby redefining how these interconnected diseases may be pharmacologically addressed.

The metabolic efficacy of **Y5** can be explained by a unified mechanism centered on enhanced hormone sensitivity rather than supraphysiological hormone signaling. PTPN1 and PTPN2 act as convergent intracellular brakes on both insulin and leptin pathways, attenuating IRS-AKT and JAK2-STAT3 signaling. Dual inhibition of these phosphatases by **Y5** restores signal transduction under resistant conditions, leading to improved insulin responsiveness, reduced compensatory hyperinsulinaemia, and reactivation of leptin-mediated regulation of energy balance. This mechanism accounts for the coordinated improvement in glycemic control, reduction in adiposity, and preservation of food intake observed across models, and distinguishes **Y5** from incretin-based agents that primarily act through central satiety pathways.

Notably, the selective PTPN1 inhibitor trodusquemine (MSI-1436) has been reported to induce appetite-independent fat-specific weight loss in DIO mice.^33^ This observation, together with our findings that **Y5** (a dual PTPN1/N2 inhibitor) also induces weight loss without reducing food intake, suggests that PTPN1 inhibition alone may be sufficient for certain appetite-independent metabolic effects. However, direct comparisons between trodusquemine and **Y5** are complicated by their distinct chemical scaffolds, pharmacokinetic profiles, and potential off-target effects. Future studies using selective inhibitors or genetic models will be needed to dissect the individual roles of PTPN1 and PTPN2 in energy balance regulation, and to determine whether dual inhibition offers advantages beyond PTPN1-selective targeting.

An important mechanistic insight of this study is the context-dependent but indispensable role of PTPN2 in mediating the metabolic effects of dual phosphatase inhibition. While PTPN1 predominantly constrains insulin receptor signaling, PTPN2 functions as a critical negative regulator of leptin sensitivity through dephosphorylation of STAT3 downstream of JAK2. In diet-induced obesity, leptin resistance arises primarily from impaired STAT3 activation rather than loss of signaling components, rendering inhibition of PTPN1 alone insufficient to restore leptin responsiveness. The moderate potency against PTPN2 is consistent with the concept that sustained, partial inhibition of this phosphatase is sufficient to relieve chronic suppression of STAT3 signaling *in vivo*.

**Y5** treatment restores STAT3 phosphorylation in cell lines and induces pronounced weight loss in diet-induced obese mice without reducing food intake, indicating reactivation of leptin signaling rather than appetite suppression. Notably, effective engagement of the leptin axis does not require equivalent inhibitory potency against PTPN2 compared with PTPN1. Instead, sustained, partial inhibition of PTPN2 enabled by the long-acting pharmacokinetic profile of **Y5** appears sufficient to relieve chronic suppression of STAT3 signaling. This principle of functional convergence rather than equal potency explains how dual PTPN1/PTPN2 inhibition simultaneously corrects insulin and leptin resistance *in vivo*.

Several limitations of the current study warrant consideration. First, although **Y5** demonstrates robust efficacy in rodent models, further evaluation in nonrodent species will be required to assess long-term safety, tissue-specific target engagement and translational pharmacokinetics. Direct evidence for DNP-specific antibody binding and FcRn-mediated recycling was not obtained in this study, and the contribution of this mechanism to **Y5**’s prolonged half-life remains to be experimentally validated in the further studies. However, the DNP-hapten strategy has been extensively validated as a generalizable platform for peptide half-life extension in the literature, and our application of this strategy to an intracellular phosphatase inhibitor represents a conceptual extension of this established approach. Second, while **Y5** restores insulin and leptin signaling across multiple tissues, the relative contributions of central versus peripheral phosphatase inhibition remain to be fully delineated. Third, the molecular docking used to predict the binding mode employed a rigid-receptor model that does not fully account for protein conformational flexibility; thus, the precise binding geometry requires further validation through cocrystallization. Nevertheless, the long-acting, peptide-based strategy described here directly addresses historical barriers associated with phosphatase drug discovery and provides a tractable framework for clinical translation. Importantly, by enhancing endogenous hormone sensitivity rather than suppressing appetite, dual PTPN1/PTPN2 inhibition may offer a differentiated therapeutic profile with improved tolerability and durability compared with existing incretin-based approaches.

In summary, this study identifies dual inhibition of PTPN1 and PTPN2 as a viable and mechanistically distinct approach for the treatment of type 2 diabetes and obesity. Through long-acting peptide-mediated suppression of these intracellular phosphatases, metabolic homeostasis is restored by enhancing insulin and leptin sensitivity rather than by suppressing appetite or augmenting hormonal signaling. The resulting long-acting glycemic control and weight loss observed in multiple mouse models support a conceptual shift in how metabolic disease can be therapeutically addressed. However, direct measurements of energy expenditure (e.g., oxygen consumption, CO_2_ production) and thermogenic markers were not performed. While unchanged food intake indicates that appetite suppression is not the primary driver, the precise contribution of increased energy expenditure to **Y5**-induced weight loss remains to be determined.

Together, our findings establish PTPN1/PTPN2 inhibition as a nonincretin strategy that directly targets the molecular basis of insulin and leptin resistance, offering a promising alternative framework for durable metabolic therapy.

## Experimental Section

### Peptide Synthesis and Circular Dichroism (CD) Spectroscopy

All BimBH3 analogues were synthesized employing standard solid-phase peptide synthesis (SPPS) with the Fmoc strategy on 2-chlorotrityl chloride (2-Cl-CTC) resin in glass reaction vessels, following established protocols. The synthesis commenced with the assembly of the decapeptide backbone sequence. Subsequently, a coupling reaction was performed using DNP-modified amino acids Fmoc-X­(DNP)-OH (X = Lys, Dab, Dap, Orn) at 4 equiv relative to the resin, 4.8 equiv of HBTU, HOBt, and DIEA in 5 mL of DMF. This reaction proceeded at 40 °C under a nitrogen atmosphere for 1 h. The resin was then thoroughly washed with DMF (4×), and the coupling efficiency was verified by the Kaiser test. In cases where the test indicated complete coupling, the synthetic steps used for the decapeptide were repeated to extend the peptide chain at the N-terminus by successively coupling Ile and a fatty acid (either octadecanedioic acid or hexadecanoic acid), employing the same coupling reagents and procedures as described for the DNP-modified amino acids.

For the synthesis of the Lys^9^-fluorescein (Flu)-labeled analogue (**Lys^9^-Flu-Y5**), Fmoc-Lys­(Alloc)-OH was incorporated following the assembly of the linear peptide chain on the resin. The Alloc protecting group was then selectively removed via two 20 min treatments with 0.1 equiv of Pd­(PPh_3_)_4_ in DCM. Finally, fluorescein labeling was achieved by coupling 5-carboxyfluorescein (5-FAM) under the Fmoc strategy for 6 hours under nitrogen protection and in the absence of light. Detailed procedures for peptide synthesis, labeling, and purification are provided in the Supporting Information.

CD spectra of the selected analogues were recorded on a Jasco J-810 spectropolarimeter over a wavelength range of 180–260 nm, using peptide concentrations of 0.2–0.6 mg/mL. Measurements were carried out in a 1.0 mm path length cell with a bandwidth of 1.0 nm, a response time of 2 s, and an average of 3 accumulations. All spectra were acquired at room temperature under a nitrogen atmosphere and are expressed as mean residue molar ellipticity [θ], calculated according to the equation

[θ]=1000×mdeg/(lc)

where mdeg is the raw CD signal in millidegrees, *c* is the peptide molar concentration (mM), and *l* is the cell path length (mm).

### Assay of Enzyme Inhibitory Activity *In Vitro*

The inhibitory activities of peptide mimetics against recombinant human PTPN1 (Sino Biological Inc., catalog no.: 10304-H07E) and PTPN2 (Sino Biological Inc., catalog no.: 10570-HNCB) were evaluated by monitoring the hydrolysis of *para*-nitrophenyl phosphate (pNPP). Peptide analogues were dissolved in dimethyl sulfoxide (DMSO) and aliquoted into a transparent 96-well polystyrene plate. Subsequently, MES buffer, pNPP, and the respective phosphatase were added to each well. The enzymatic reaction was performed at room temperature, and the catalysis of pNPP was continuously monitored at 405 nm for 4 min. Dose–response curves for each analogue were generated, and the half-maximal inhibitory concentration (IC_50_) was calculated using GraphPad Prism 8.0 (GraphPad Software Inc.).

### Molecular Docking Studies

The crystal structures of PTPN1 (PDB ID: 9CYO, resolution: 1.94 Å) and PTPN2 (PDB ID: 7UAD, resolution: 2.04 Å) were retrieved from the Protein Data Bank (PDB) and used as receptor models.. Prior to docking, both receptor and ligand structures were preprocessed by removing water molecules and heteroatoms, followed by protonation and energy minimization to obtain the most stable conformations. Semiflexible molecular docking was performed using AutoDock Vina (version 1.1.2). Systematic sampling of ligand positions, orientations, and rotatable bonds was conducted within the defined active site region. Multiple rounds of efficient search were performed using the scoring function to identify the ligand conformation with the lowest binding free energy. The docking results obtained from AutoDock Vina were visualized using PyMOL (version 2.0, Schrödinger, LLC) to analyze specific binding interactions, such as hydrogen bonds and hydrophobic interactions.

### Molecular Dynamics Simulations

All molecular dynamics (MD) simulations were performed using GROMACS (version 2023.3). The system was solvated with explicit water molecules and neutralized by adding counterions. Long-range electrostatic interactions were treated with the particle mesh Ewald (PME) method, and both electrostatic and van der Waals interactions were cutoff at 1.0 nm. Energy minimization was first carried out using the steepest descent algorithm until the maximum force dropped below 1000 kJ mol^–1^ nm^–1^. The system was then equilibrated in two steps: (i) a 100 ps NVT ensemble at 300 K using a V-rescale thermostat, and (ii) a 100 ps NPT ensemble at 1 bar using a Berendsen barostat. During equilibration, heavy atoms were position-restrained. Finally, a 50 ns production MD run was conducted under NPT conditions (300 K, 1 bar). All simulations were accelerated with mixed-precision calculations on GPU nodes (NVIDIA A100).

### DPP-IV Stability

To evaluate the stability of the peptide mimetics against DPP-IV enzyme, a stock solution (1 mg/mL) was prepared by dissolving the peptide in sodium phosphate buffer (20 mM, pH 7.0). The reaction was initiated by adding DPP-IV (4.5 μL, ≥45 U/mg) to the solution, followed by incubation at 37 °C. At designated time points, 100 μL aliquots were withdrawn and quenched with 200 μL of 0.1 M sodium bicarbonate. The remaining peptide content was analyzed by RP-HPLC at 214 nm. Peak areas corresponding to the peptide at different time points were integrated, and the data were fitted using GraphPad Prism 8 to determine the half-life by plotting the residual peptide amount versus time.

### Plasma Stability

The plasma stability of the peptide mimetics was assessed by adding 200 μL of the peptide solution (0.01–0.04 M in phosphate-buffered saline, PBS, pH 7.4) to 200 μL of 40% mouse plasma (Lot no. MP20039, Shanghai Yuanye Bio-Technology Co., Ltd.) at 37 °C. At predetermined time points (0, 0.5, 1, 3, 10, 16, 22, and 40 h), 20 μL aliquots were rapidly withdrawn and immediately quenched with an equal volume of 0.2% TFA in acetonitrile. The resulting mixture was centrifuged at 14,000 rpm for 10 min at 4 °C to remove precipitated proteins. The supernatant was subsequently injected into an analytical HPLC system and analyzed at 214 nm. The residual peptide content at each time point was calculated based on the integrated peak area, and the data were imported into GraphPad Prism 8. The half-life was determined by fitting the percentage of remaining peptide versus time.

### Cell Culture and Western Blot

HepG2 cells were cultured in Minimum Essential Medium (MEM) supplemented with 10% (v/v) fetal bovine serum, 100 μg/mL streptomycin, and 100 U/mL penicillin. The cells were maintained at 37 °C in a humidified incubator with an atmosphere of 95% air and 5% CO_2_. For the experiments, cells were seeded in 6-well plates at a density of 5 × 10^5^ cells per well and allowed to reach 60–70% confluence. To induce insulin or leptin resistance, the cells were treated with 10^–6^ mol/L insulin and/or 100 μmol/L palmitic acid (PA) for 24 h. Following this induction period, the medium was replaced with fresh medium containing different concentrations of **Y5**, and the cells were incubated for an additional 24 h. Subsequently, the cells were stimulated with 100 nM insulin or 100 nM leptin for 30 min.

After the treatments, the cells were washed twice with cold PBS. Total protein was then extracted using RIPA lysis buffer supplemented with protease and phosphatase inhibitors. The lysates were incubated on ice for 10 min, followed by centrifugation at 12,000 rpm for 10 min at 4 °C to collect the supernatant. The protein concentration of the supernatants was determined using a BCA assay kit. Equal amounts of protein from each sample were separated by 8% or 12.5% SDS-PAGE and transferred PVDF membranes. The membranes were blocked with 5% nonfat milk in TBST for 1 h at room temperature to prevent nonspecific binding. Thereafter, the membranes were incubated with the appropriate primary antibodies overnight at 4 °C, followed by incubation with horseradish peroxidase (HRP)-conjugated secondary antibodies for 1 h at room temperature. Protein bands were visualized using an infrared laser imaging system.

### Pharmacokinetic Evaluation

The fluorescence-based PK method has been validated in previous work by Dai et al.,^45^ where fluorescence-labeled analogues were used for PK studies with results validated by LC-MS/MS (verification via cross-study data). Prior to administration, Sprague-Dawley (SD) rats were weighed and received a single subcutaneous injection of the fluorescently labeled analogue **Lys^9^-Flu-Y5** at a dose of 100 nmol/kg. At predetermined time points postinjection, blood samples (80 μL) were collected from the tail vein and mixed with 320 μL of PBS containing EDTA (pH 8.0). The blood samples were centrifuged at 3000 rpm for 15 min at 4 °C to separate plasma. The resulting plasma samples were then analyzed using a multimode microplate reader (synergy H4, Biotek) with excitation and emission wavelengths set at 490 and 520 nm, respectively. Plasma concentration–time profiles were generated using GraphPad Prism 8, and pharmacokinetic parameters were estimated with Phoenix WinNonlin software (Academic Version, Pharsight Corp., Mountain View, CA, USA).

### Biodistribution Analysis

The temporal distribution profile of **Lys^9^-Flu-Y5** was evaluated in Kunming mice divided into two groups (*n* = 3 per group) following subcutaneous administration of the compound at a dose of 100 nmol/kg. Mice were maintained in the dark postinjection to minimize photobleaching and anesthetized with isoflurane at scheduled time points (0, 0.5, 1, 2, and 4 h). Whole-body fluorescence imaging was conducted using a small animal *in vivo* imaging system (FMT 2500; PerkinElmer, Waltham, MA, USA) with an excitation wavelength of 520 nm. Imaging data were quantitatively analyzed using TrueQuant software (version 3.0).

### Antidiabetic and Antiobesity Efficacy in db/db and Diet-Induced Obese Mice

All experimental procedures involving animals were reviewed and approved by the Laboratory Animal Ethics Committee of Ocean University of China (approval no. SYXK (Shandong) 20210009). The study was conducted at the Marine Biomedical Research Institute of the university, in strict accordance with the NIH Guide for the Care and Use of Laboratory Animals (eighth edition, 2011), under the oversight of the Department of Science and Technology of Shandong Province.

Male db/db mice (8–9 weeks old) were housed under standard conditions (24 ± 2.0 °C, normal light-dark cycle) with bedding and purified water provided, which was refreshed every 2 days. After a one-week acclimation period, mice exhibiting fasting blood glucose levels exceeding 11 mM were selected and randomly divided into five groups (*n* = 5 per group): a vehicle control, a positive control, and three dose-groups (high, medium, low) of test compound **Y5**. All groups received once-weekly subcutaneous injections: the positive control was administered semaglutide, the **Y5** groups received respective doses of compound **Y5**, and the vehicle control received an equal volume of physiological saline. Blood glucose levels were monitored twice per week via tail vein sampling using an ACCU-CHEK Performa glucometer (Roche Diabetes Care GmbH, Mannheim, Germany). Body weight was measured twice weekly, while food and water consumption were recorded daily. In a parallel study using diet-induced obese (DIO) mice, housing conditions were similar. These mice received subcutaneous administration of compounds once every 3 days, with body weight and food intake monitored daily throughout the treatment period.

### Glucose Tolerance Test (GTT)

The area under the curve (AUC) for blood glucose was calculated from actual measured values following an oral glucose tolerance test (OGTT), which was performed to assess the ability of the peptides to restore normoglycemia. After an overnight fast, mice received a subcutaneous (s.c.) injection of the designated peptides, semaglutide, or vehicle, prior to an oral glucose challenge (2 g/kg). Blood glucose was measured immediately before the glucose load (t = 0) to establish any peptide-induced alterations in baseline levels, and subsequently at 30, 60, 90, and 120 min postadministration.

### Biomarker Analysis

Fresh serum samples were analyzed directly after gentle mixing to aliquot the supernatant for testing. Prior to instrumental analysis, sample volume was verified to be sufficient for all intended assays. The required sample volume ranged from 2 to 35 μL per analyte, with cumulative consumption increasing with the number of biomarkers assessed. In cases of limited sample volume or high-plex assays, samples were appropriately diluted at a predetermined factor. Notably, significant hemolysis was considered a potential interference in assay accuracy. The biomarkers were measured using enzyme-linked immunosorbent assay (ELISA) kits.

### Statistical Analysis

Statistical analyses were performed using GraphPad Prism. Comparisons between multiple groups at a single time point were conducted using one-way ANOVA followed by Tukey’s multiple comparisons test. Changes over time between treatment groups were analyzed by two-way ANOVA with treatment and time as factors. Data are presented as the mean ± SD, and individual data points represent biological replicates (individual mice). A two-tailed *p*-value of <0.05 was considered statistically significant. Data distribution was assumed to be normal and was not formally tested.

## Supplementary Material



## Notes

Additional details for experimental procedures, peptide purity and MS data of BimBH3 analogues, raw Western blot images, and HPLC and MS characterizations of peptides (PDF)
