# Supplementary material for: The PTPN1/N2 Peptide Inhibitor Achieves Potent, Long-Acting Glycemic Control and Weight-Loss Dual Efficacy in Mice
Source: ACS Omega. 2026 Sep 23;11(39):59556–71. doi: 10.1021/acsomega.6c05930 (PMC13647951; doi:10.1021/acsomega.6c05930)

Supplementary Information

The PTPN1/N2 Peptide Inhibitor Achieves Potent, Long-acting Glycemic Control and Weight-loss Dual Efficacy in Mice

Yiying Shi[1], Xianmin Yang[1], Fanxu Meng[3], Anton Feenstra[3], Jin Chen[2], Zongwen Gu[1], Xiang Gao[1], Wenqing Yao[1], Guozhen Dong[1], Liyan Gong[1], Han Wu[2], Chuanliang Zhang[1, 2,*], Yuchao Gu[2,4,*]

[1.] *Qingdao Key Laboratory of Biomacromolecular Drug Discovery and Development, College of Chemical Engineering, Qingdao University of Science and Technology, Qingdao 266042, China;*

[2.] *Qingdao Center of Technology Innovation for Shark Antibody Development, College of Biological Engineering, Qingdao University of Science and Technology, Qingdao 266042, China;*

[3.] *Department of Computer Science, Vrije Universiteit Amsterdam, 1081HV, Amsterdam, The Netherlands.*

[4.] *School of Medicine and Pharmacy, Ocean University of China, Qingdao 266003, China.*

*To whom correspondence should be addressed: zhangcl@qust.edu.cn (C. L. Zhang), guych@126.com (Y. C. Gu).

Contents of SI

1. Peptide Synthesis and Purification and Inhibitory activity.

All analogues were synthesized manually via standard Fmoc-based solid-phase peptide synthesis (SPPS) using 2-Cl-CTC resin in a glass reaction vessel, following a previously reported procedure. Briefly, coupling reactions were performed by treating the resin (1 equiv) with a preactivated solution of N-α-Fmoc-protected amino acid (2 equiv), HOBT (2.4 equiv), HBTU (2.4 equiv), and DIEA (2.4 equiv) in anhydrous DMF (3–5 mL/g resin). The mixture was agitated at room temperature for approximately 1 hour. The completion of each coupling step was monitored using the Kaiser test. Fmoc deprotection was achieved by two sequential 10-minute treatments with 20% (v/v) piperidine in DMF. This cycle of coupling and deprotection was repeated iteratively until the entire peptide backbone was assembled. Subsequently, a coupling reaction was performed using DNP-modified amino acids Fmoc-X(DNP)-OH（X = Lys, Dab, Dap, Orn)at 4 equivalents relative to the resin, 4.8 equivalents of HBTU, HOBt, and DIEA in 5 mL of DMF. This reaction proceeded at 40 °C under a nitrogen atmosphere for 1 hour. The resin was then thoroughly washed with DMF, and the coupling efficiency was verified by Kaiser's test. Upon completion of the Kaiser test, the coupling of isoleucine and the fatty acids (hexadecanoic acid or octadecanedioic acid) was performed under conditions analogous to those used for Fmoc-X(DNP)（X = Lys, Dab, Dap, Orn). For the synthesis of the Lys 9-fluorescein (Flu)-labeled analog (Lys 9-FluY5), Fmoc-Lys(Alloc)-OH was incorporated following the assembly of the linear peptide chain on the resin. The Alloc protecting group was then selectively removed via two 20-minute treatments with 0.1 equivalent of $Pd(PPh_3)_4$ in DCM. **Finally,** fluorescein labeling was achieved by coupling 5-carboxyfluorescein (5-FAM) under the Fmoc strategy for 6hours under nitrogen protection and in the absence of light. Following chain assembly, the crude peptides were cleaved from the resin and simultaneously deprotected using a cleavage cocktail composed of TFA/DODT/TIS/water (92.5:2.5:2.5:2.5, v/v). The resulting solution was concentrated

under a stream of nitrogen gas. The peptides were then precipitated in cold diethyl ether, pelleted via centrifugation, and washed with fresh ether (3-5 times). The resulting pellets were subsequently re-suspended in 50-80% (v/v) acetonitrile/water. Finally, purification of the crude products was achieved by C18 reversed-phase semi-preparative HPLC. The purification was performed under the following chromatographic conditions: the mobile phase consisted of acetonitrile and water (containing 0.1% TFA), using a linear gradient from 5% to 100% acetonitrile over 40 minutes. The detection wavelength and flow rate were set at 214 nm and 10 mL/min, respectively. The target peaks were collected, and acetonitrile was removed from the combined fractions by rotary evaporation. Lyophilization of the resulting aqueous solution afforded the purified analogues **Y1-Y8** as flocculent or powdery solids. The final products were characterized by electrospray ionization-mass spectrometry (ESI-MS) and analyticalhigh-performance liquid chromatography (HPLC). The purity and mass spectrometry data for the synthesized peptide mimetics are listed in Table. The analytical HPLC trace and mass spectrogram of analogues are illustrated in the following attached **Fig. S1**-**S10**.

TableS1:BimBH3 mimics peptide purity and mass spectrometry data.

**Analogue**	**Purity (%)**	**Purity (%)**	
		**Calculated**	**Observed**
Y1	97.28	1907.05	$[M+2H]^{2+}$955.05
Y2	99.08	1865.00	$[M+2H]^{2+}$934.00
Y3	98.51	1893.04	$[M+2H]^{2+}$947.7<br>$[M+3H]^{3+}$632.3
Y4	99.97	1879.02	$[M+2H]^{2+}$940.95
Y5	99.70	1965.06	$[M+2H]^{2+}$984.05
Y6	99.59	1923.01	$[M+2H]^{2+}$963.00
Y7	98.96	1951.04	$[M+2H]^{2+}$976.70<br>$[M+3H]^{3+}$651.5
Y8	98.23	1937.02	$[M+2H]^{2+}$969.70
Y5-C18 only	96.55	1742.00	$[M-2H]^{2-}$ 870.40
Y5-DNP only	95.9	1668.82	$[M-2H]^{2-}$ 833.60

2.The Raw Figures of Western Blot for Signaling Molecules And Protein Expression Analysis Results.

p-AKT

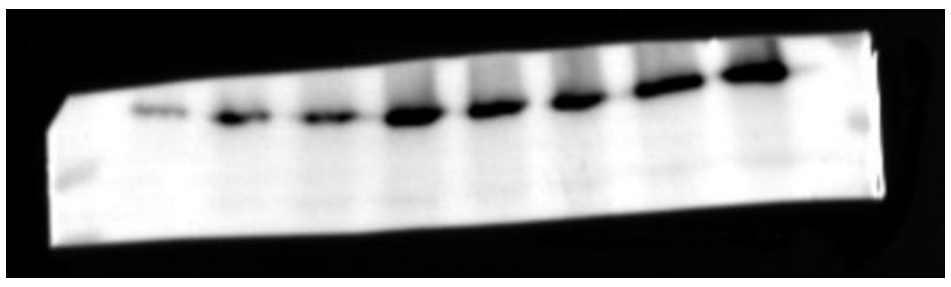

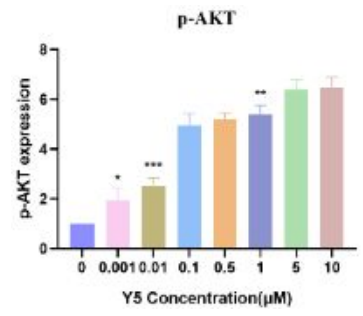

*, p<0.05; **, p<0.01; ***, p<0.001

P-IRS1

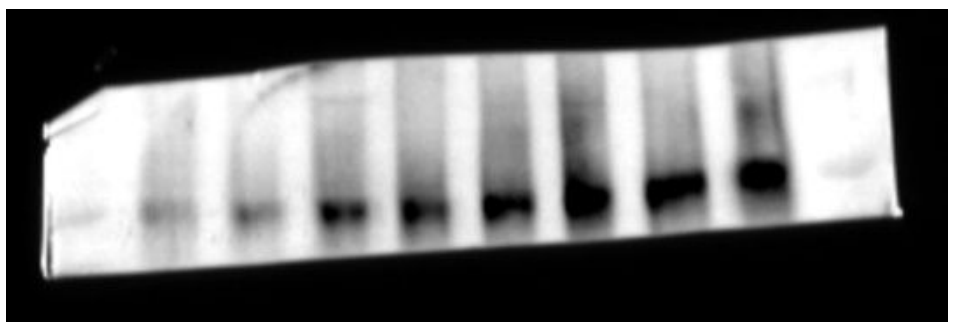

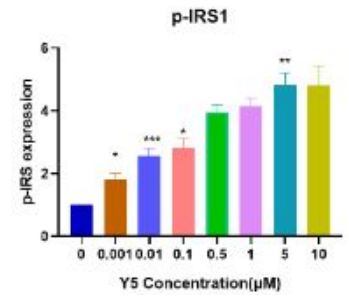

*, p<0.05; **, p<0.01; ***, p<0.001

P-STAT3

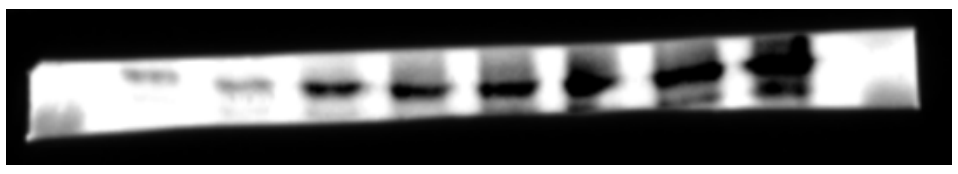

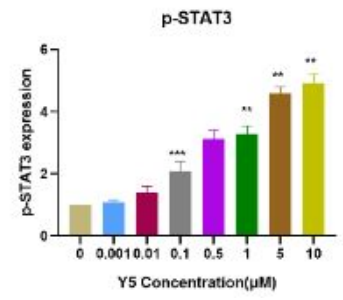

*, p<0.05; **, p<0.01; ***, p<0.001

PTPN1

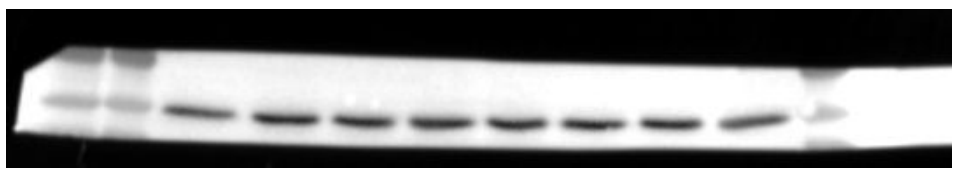

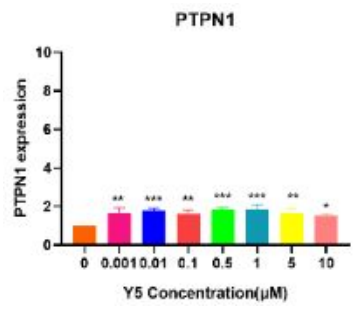

*, p<0.05; **, p<0.01; ***, p<0.001

GAPDH

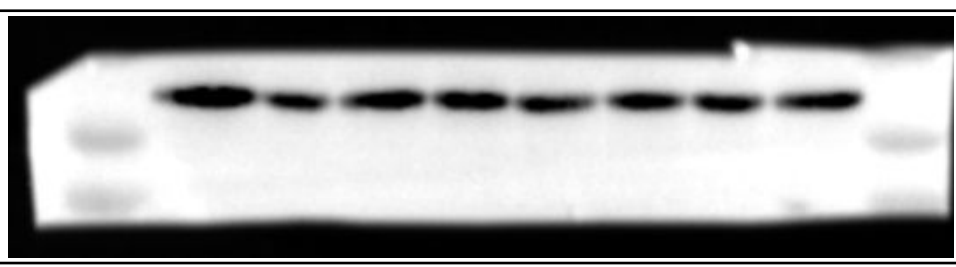

3.The analytical HPLC trace and mass spectrogram of analogues.

Fig. S1: Analytical HPLC trace (top) and mass spectrogram (bottom) of analogue Y1.

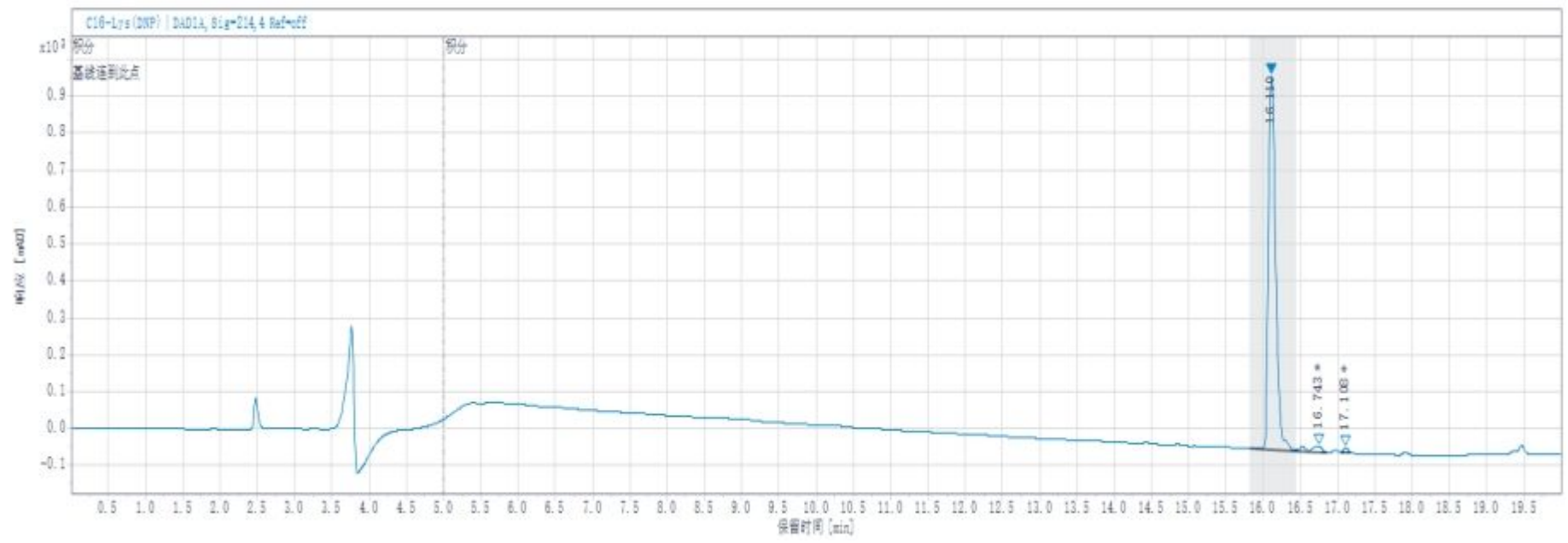

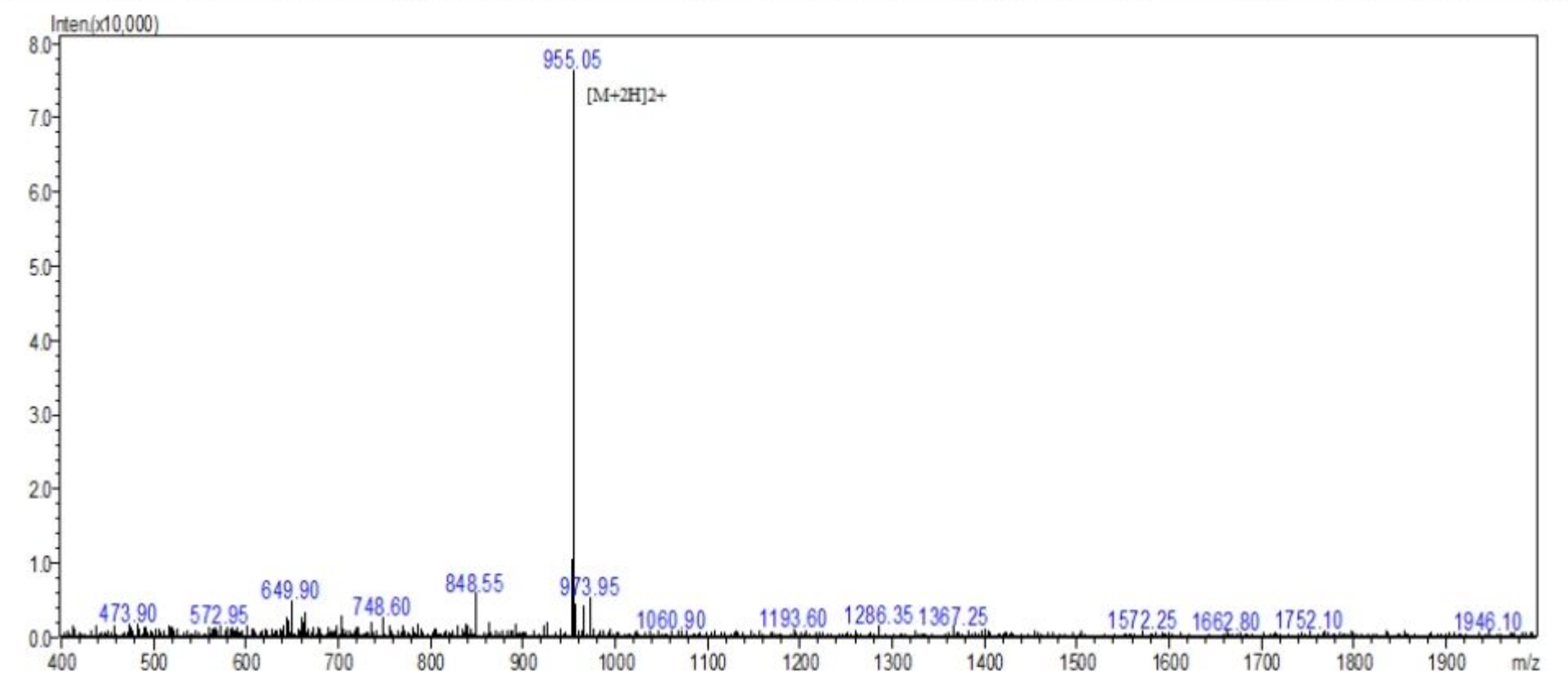

Fig. S2: Analytical HPLC trace (top) and mass spectrogram (bottom) of analogue Y2

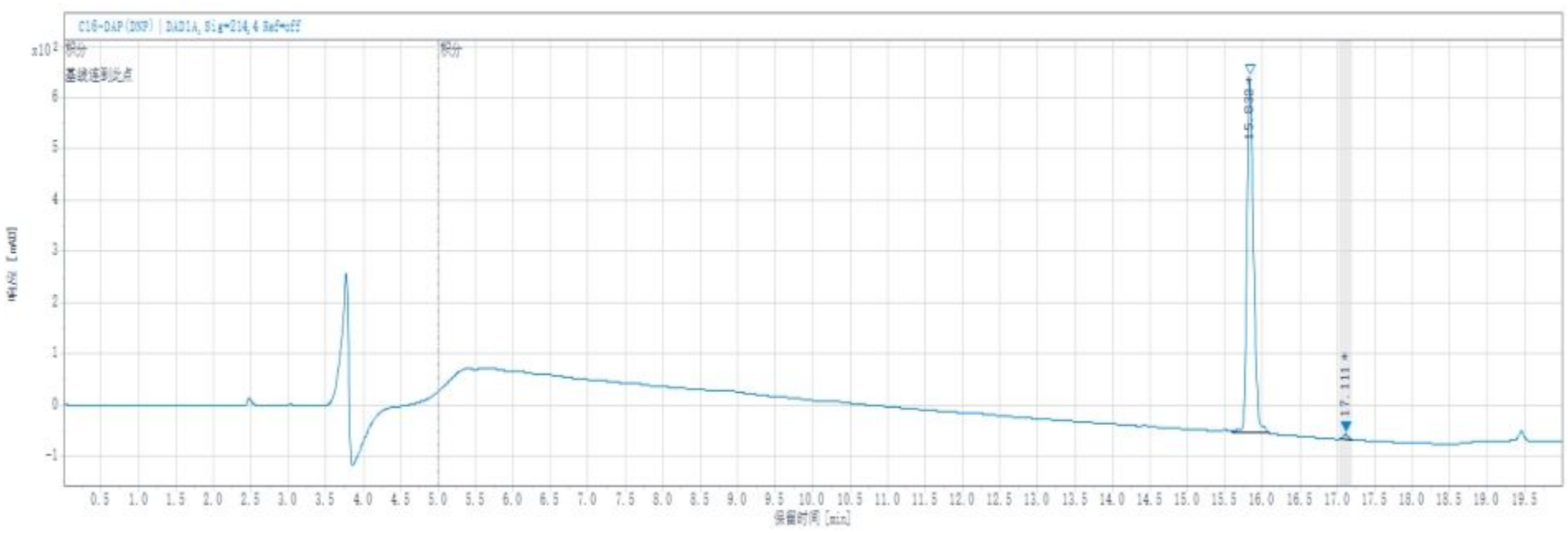

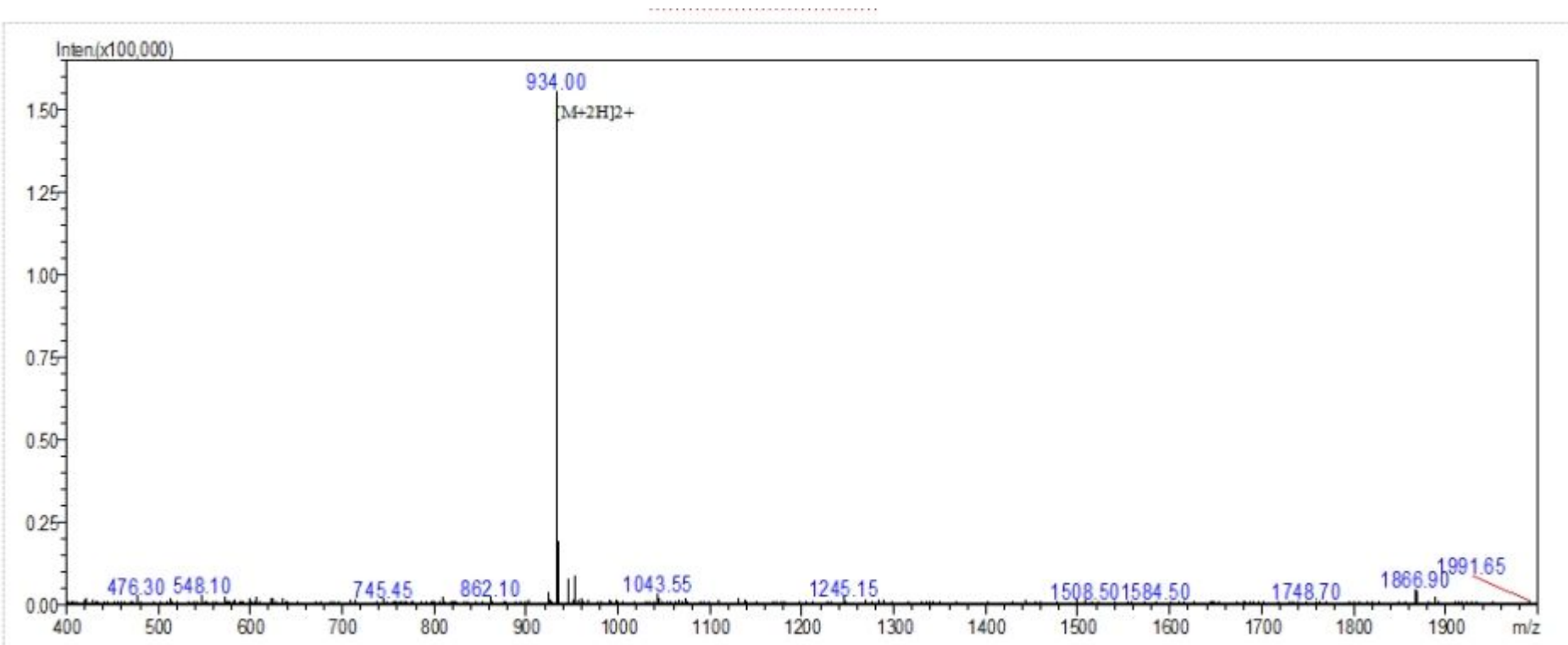

Fig. S3: Analytical HPLC trace (top) and mass spectrogram (bottom) of analogue Y3.

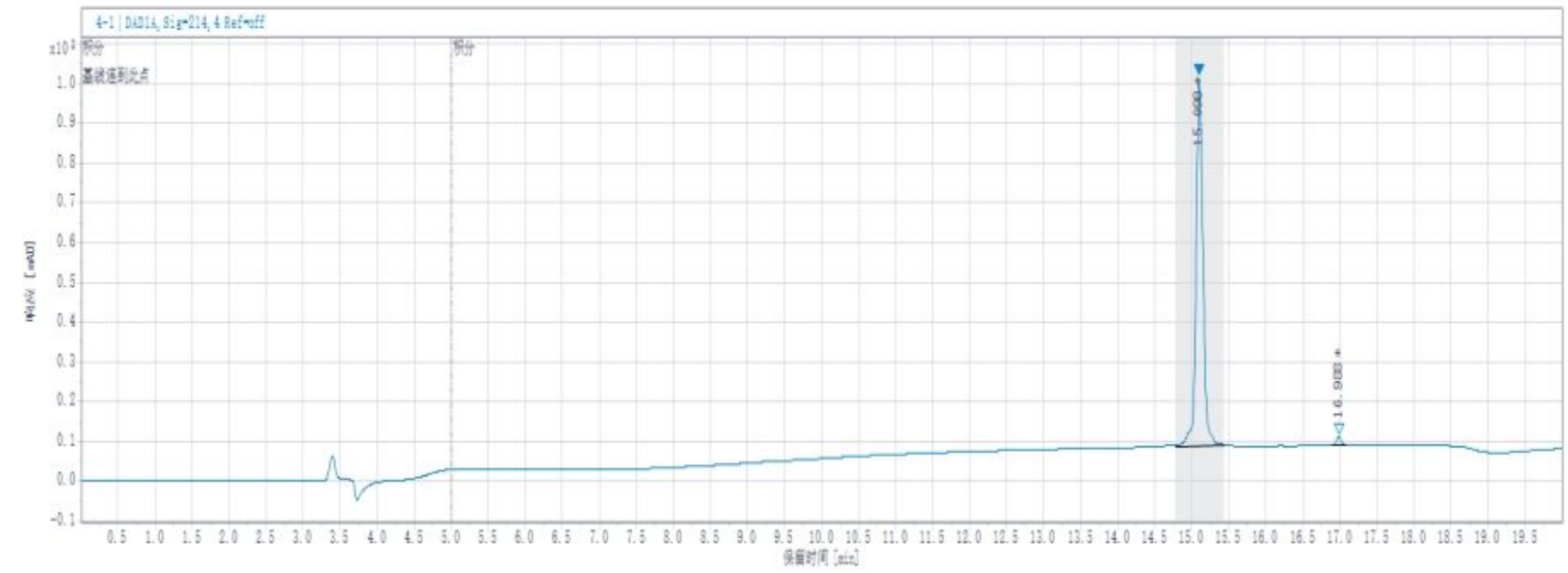

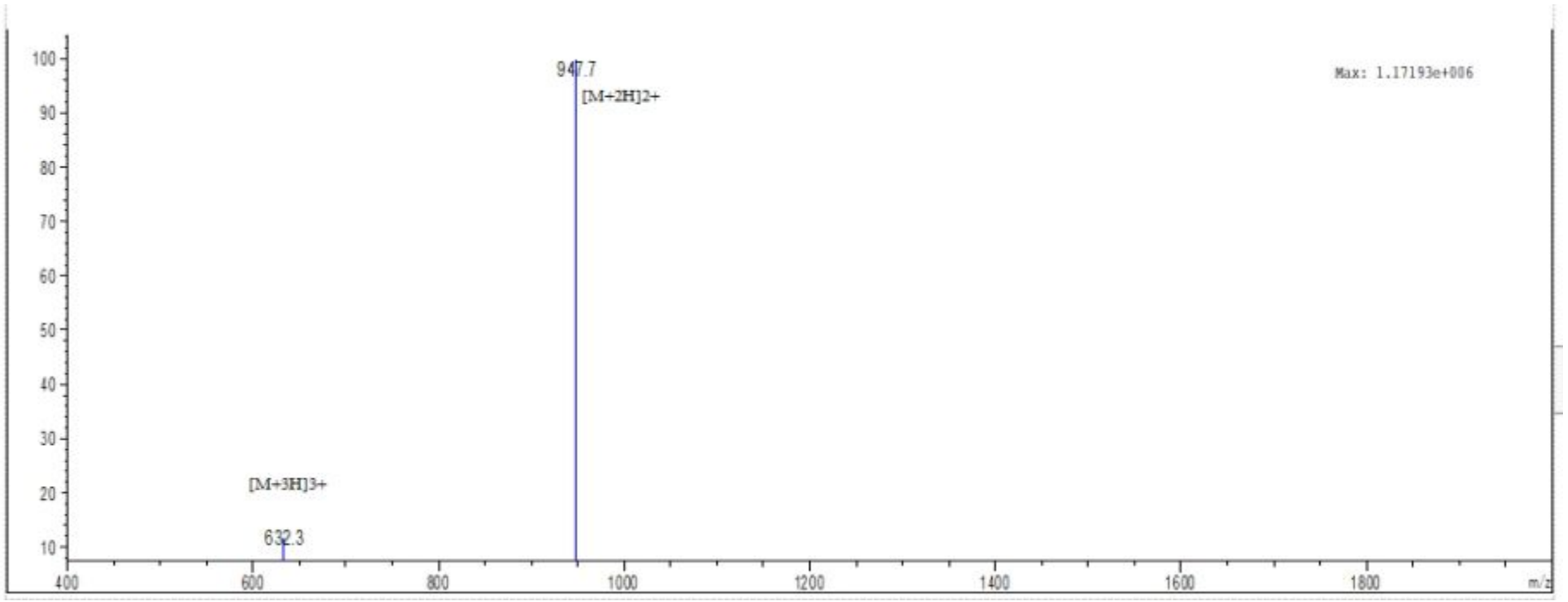

Fig. S4: Analytical HPLC trace (top) and mass spectrogram (bottom) of analogueY4

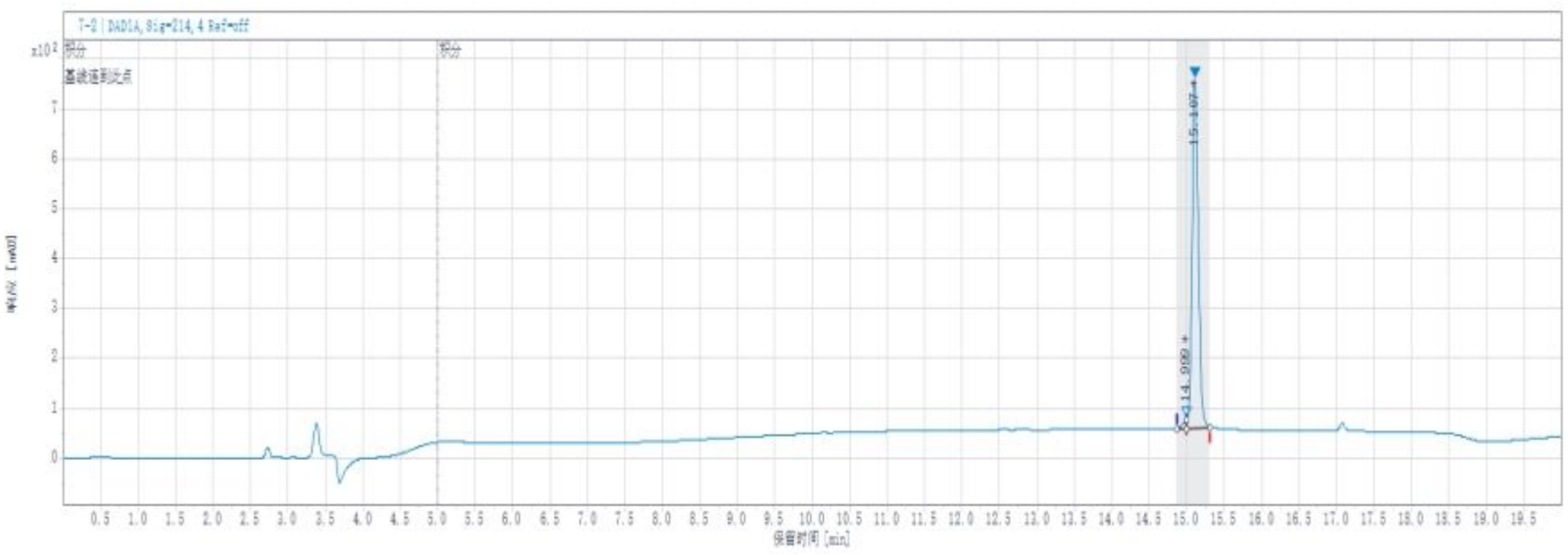

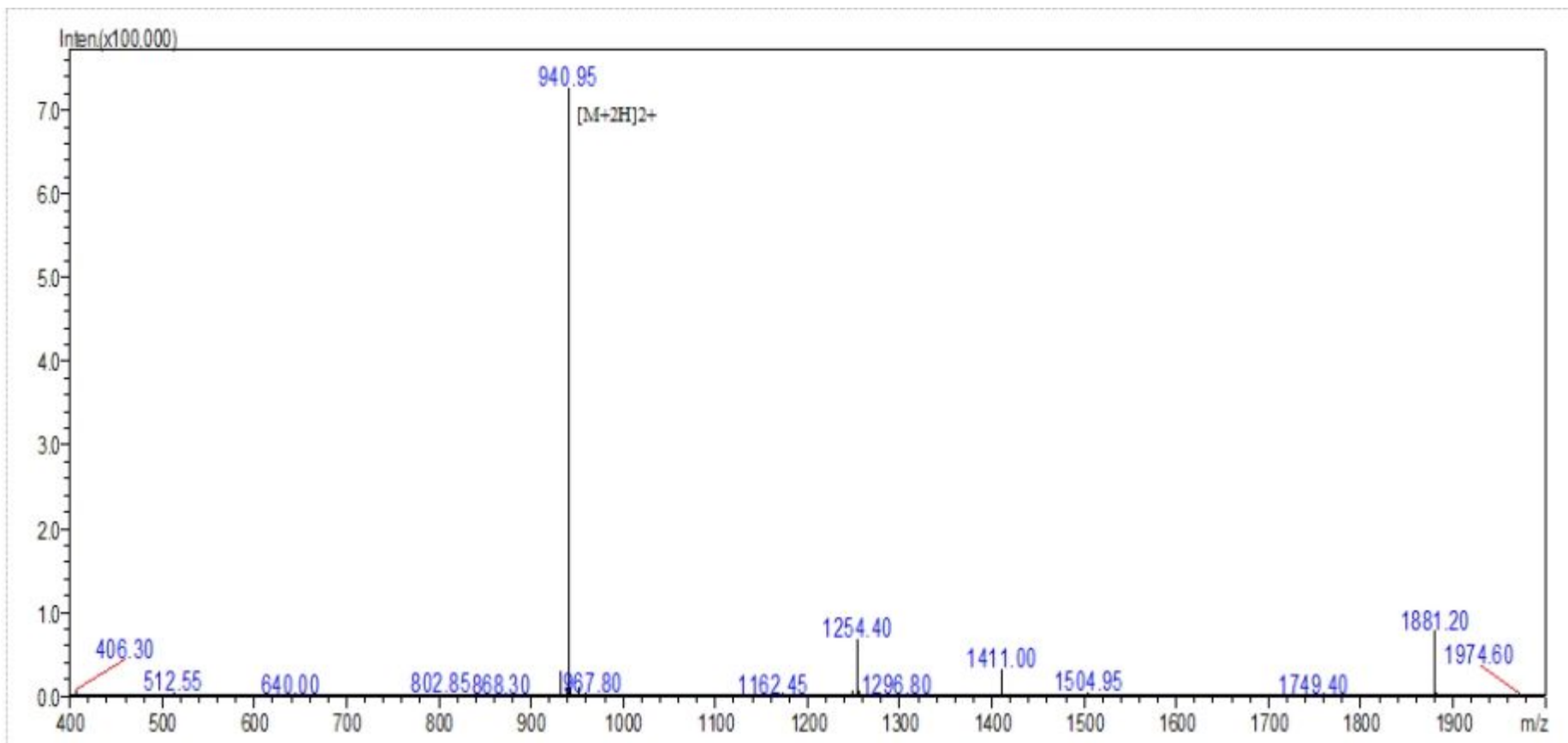

Fig. S5: Analytical HPLC trace (top) and mass spectrogram (bottom) of analogue Y5

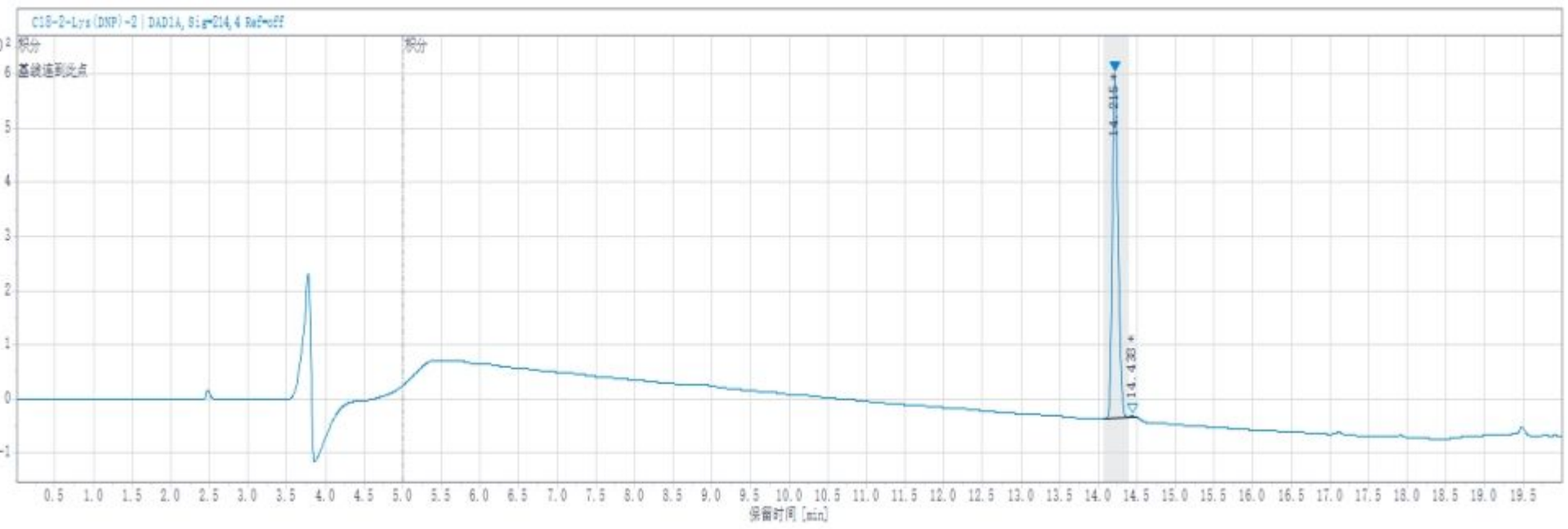

.

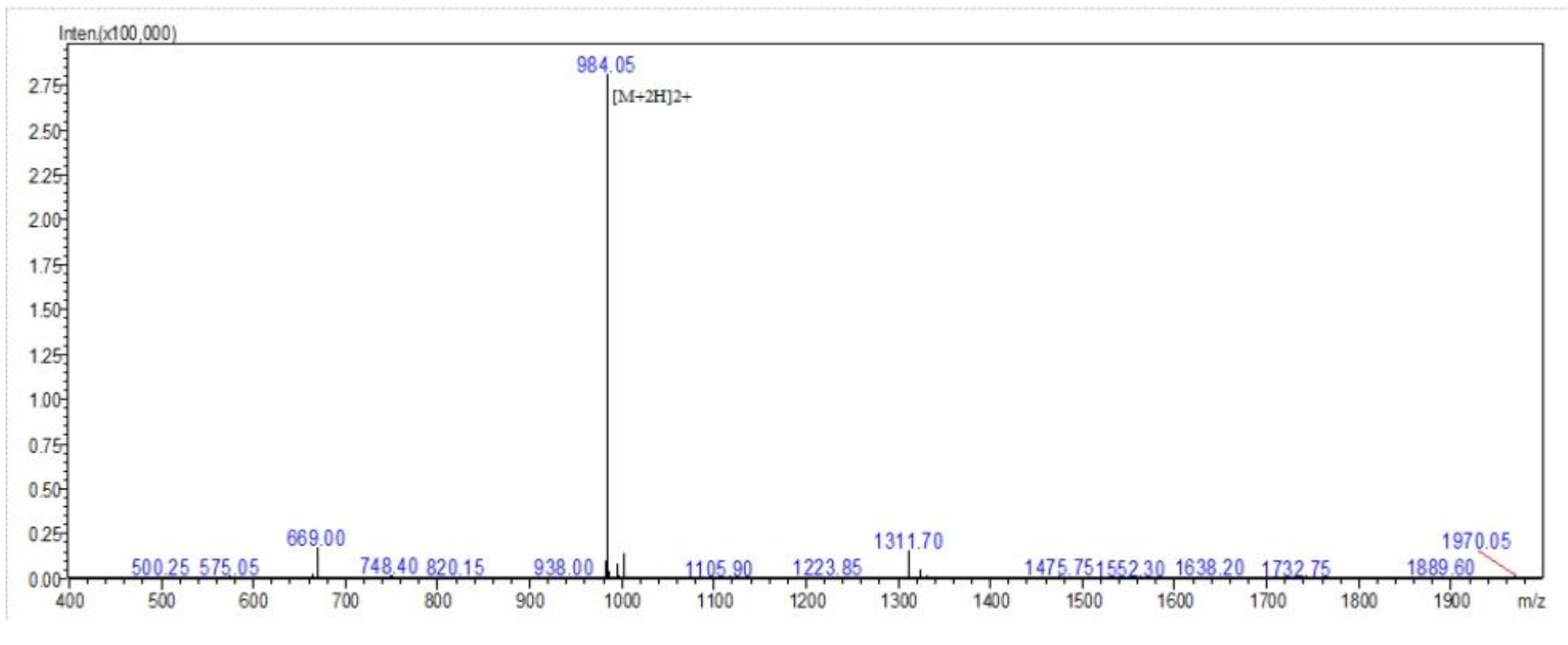

Fig. S6: Analytical HPLC trace (top) and mass spectrogram (bottom) of analogue Y6.

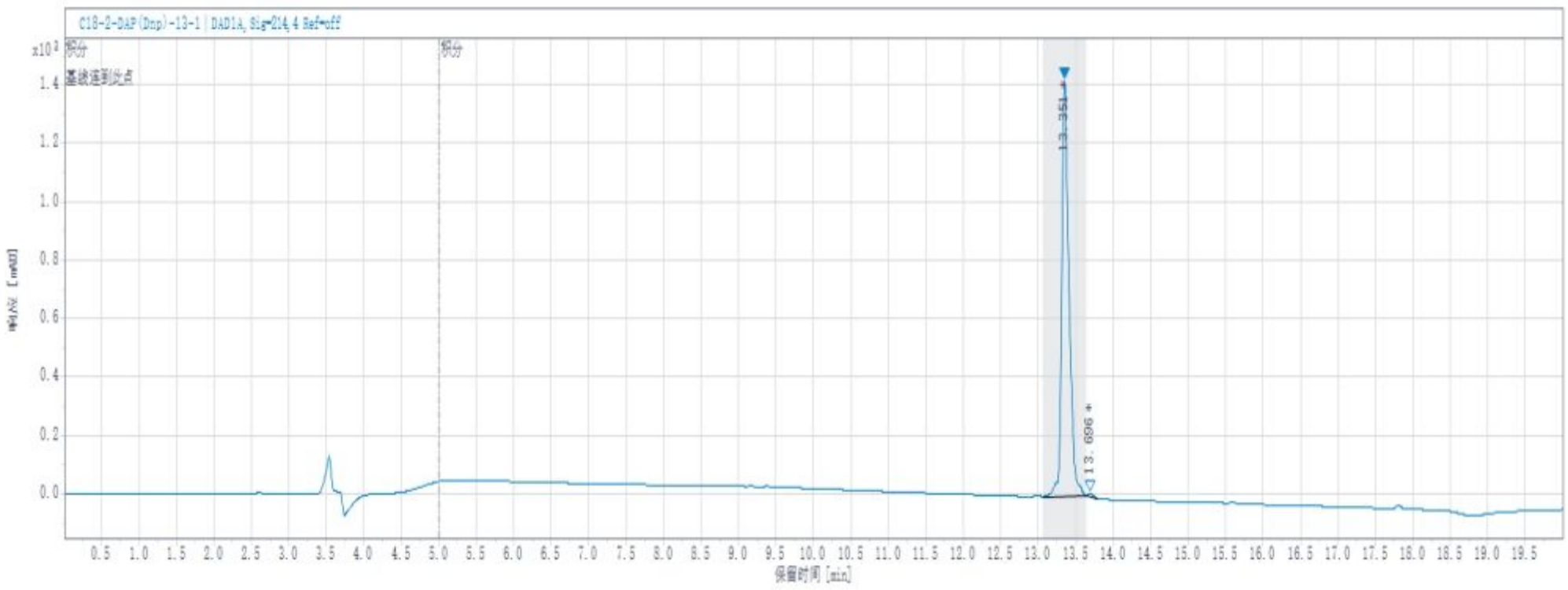

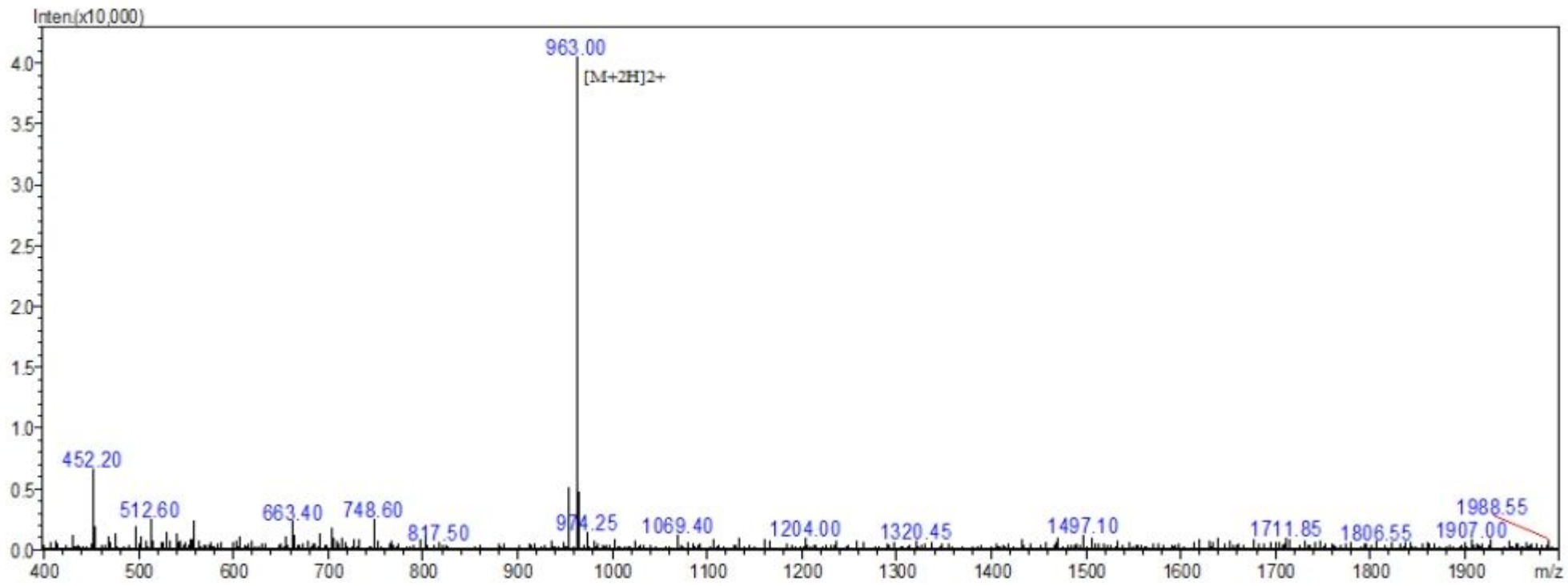

Fig. S7: Analytical HPLC trace (top) and mass spectrogram (bottom) of analogue Y7.

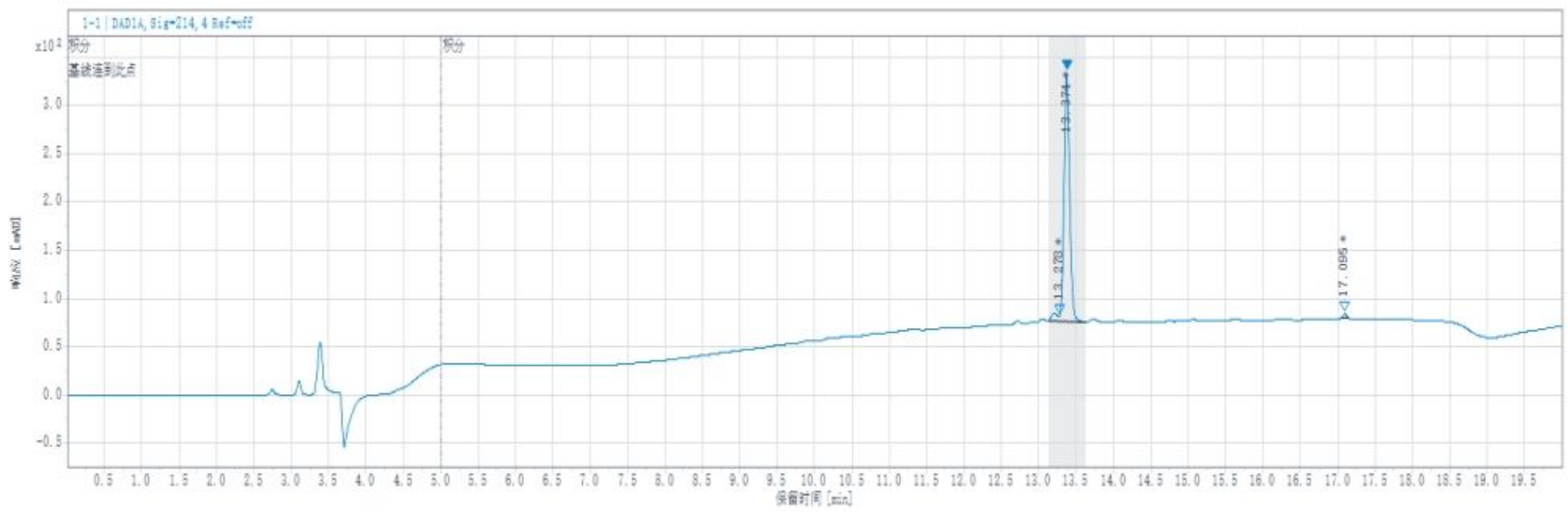

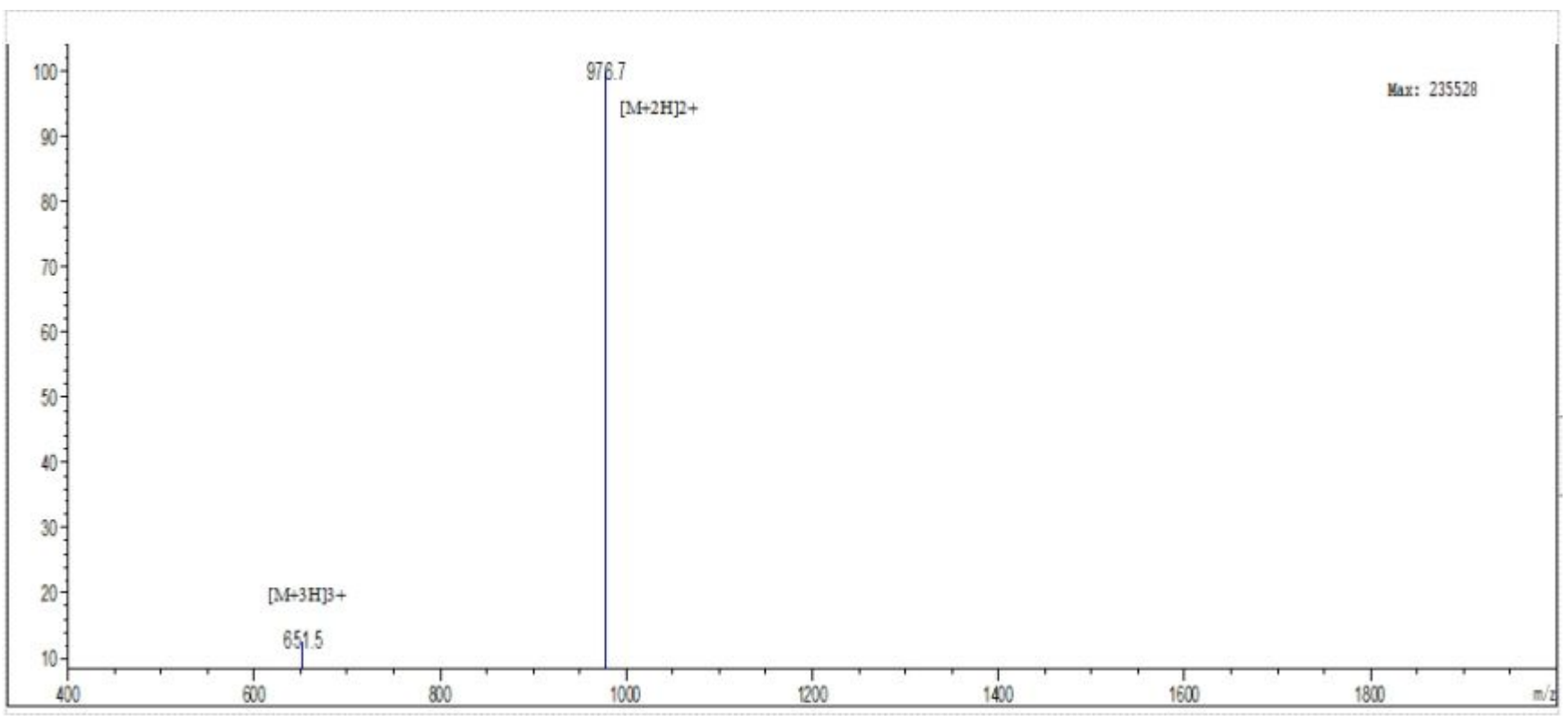

Fig. S8: Analytical HPLC trace (top) and mass spectrogram (bottom) of analogue Y8.

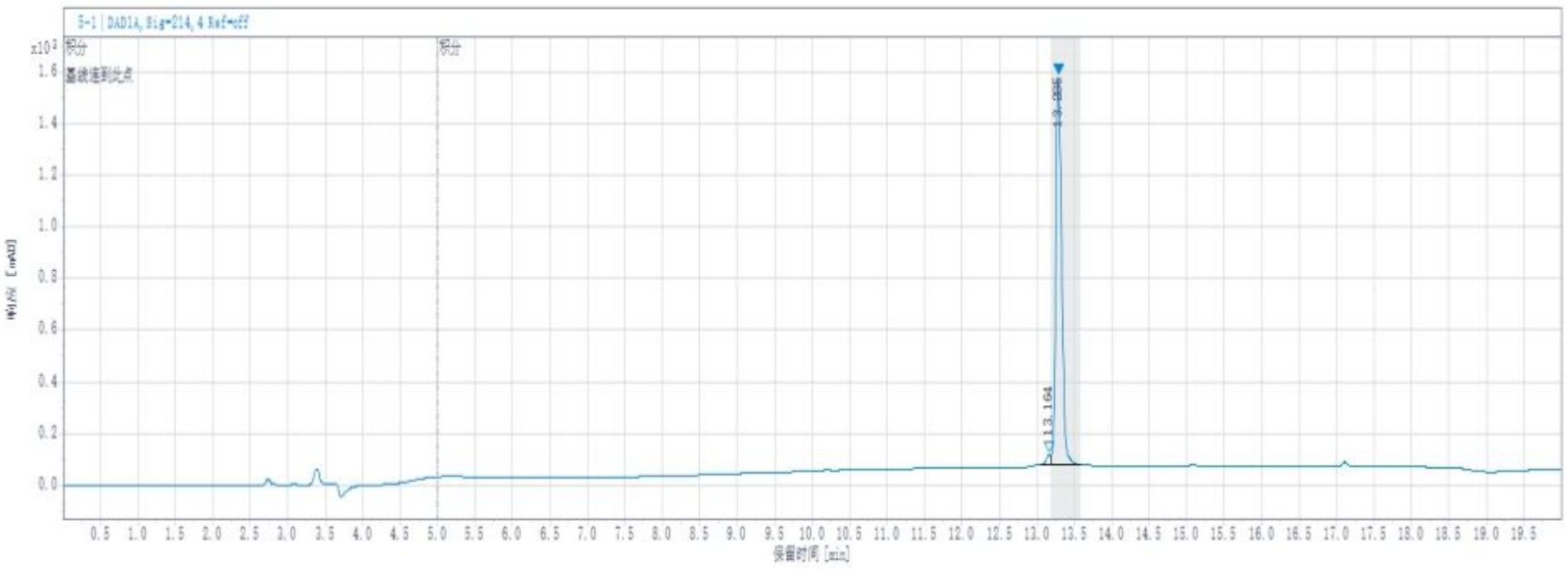

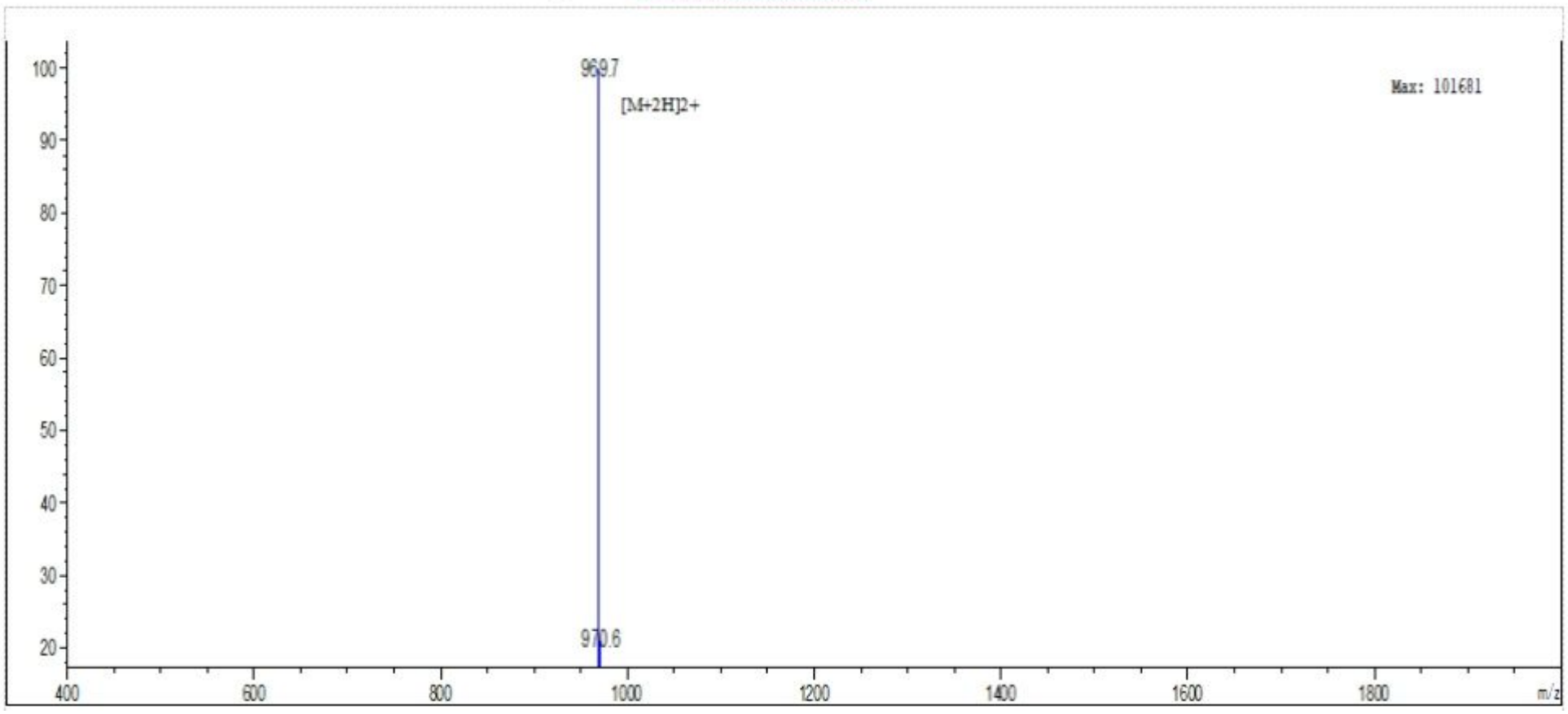

Fig. S9: Analytical HPLC trace (top) and mass spectrogram (bottom) of analogue Y5-C18 only

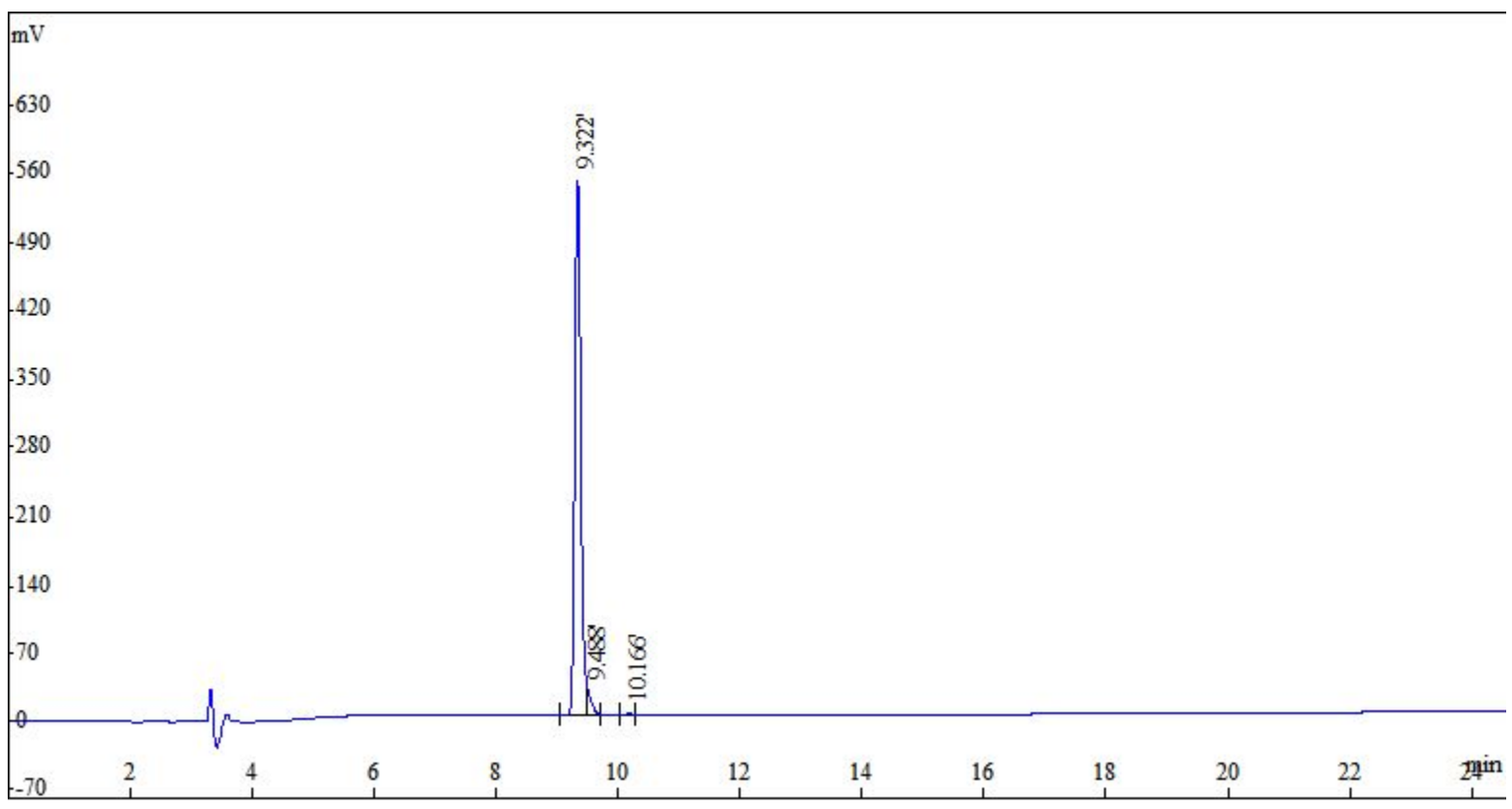

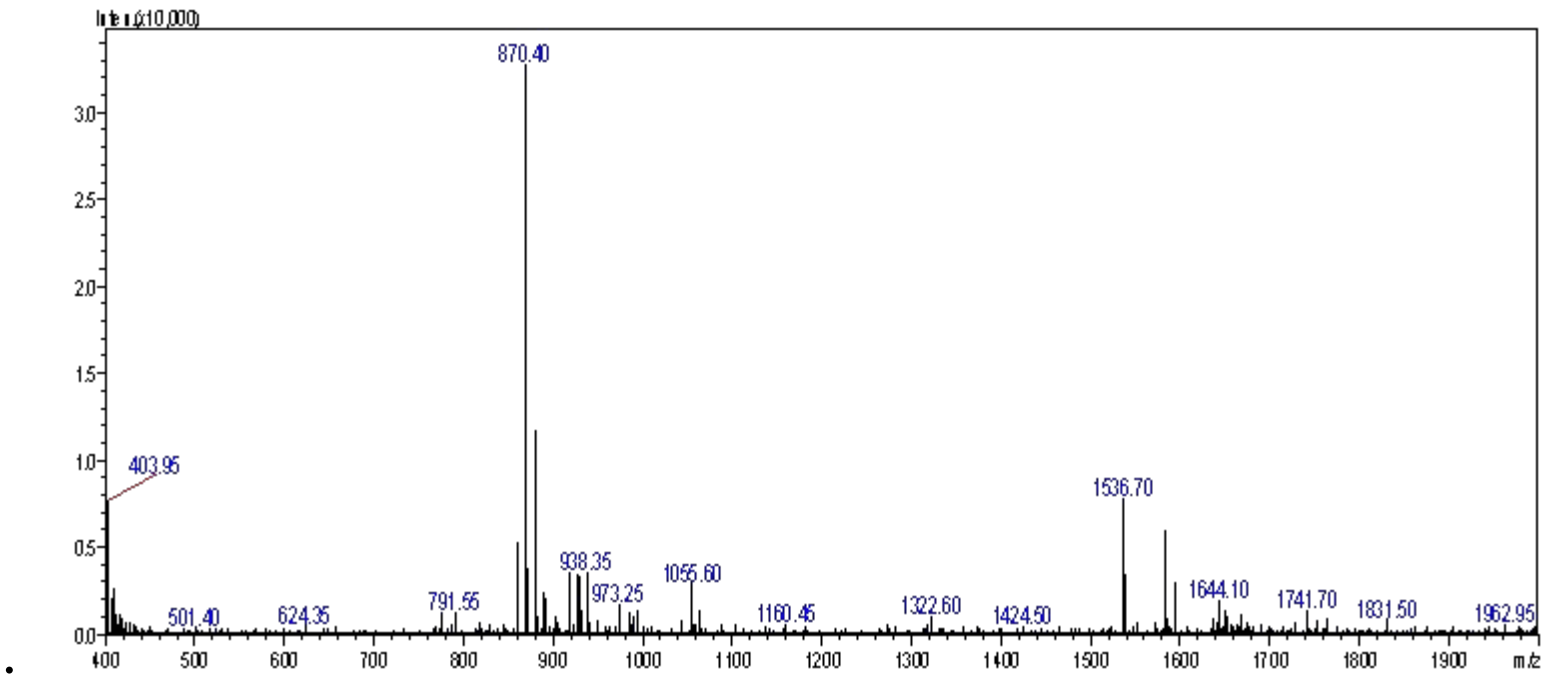

Fig. S10: Analytical HPLC trace (top) and mass spectrogram (bottom) of analogue Y5- DNP only

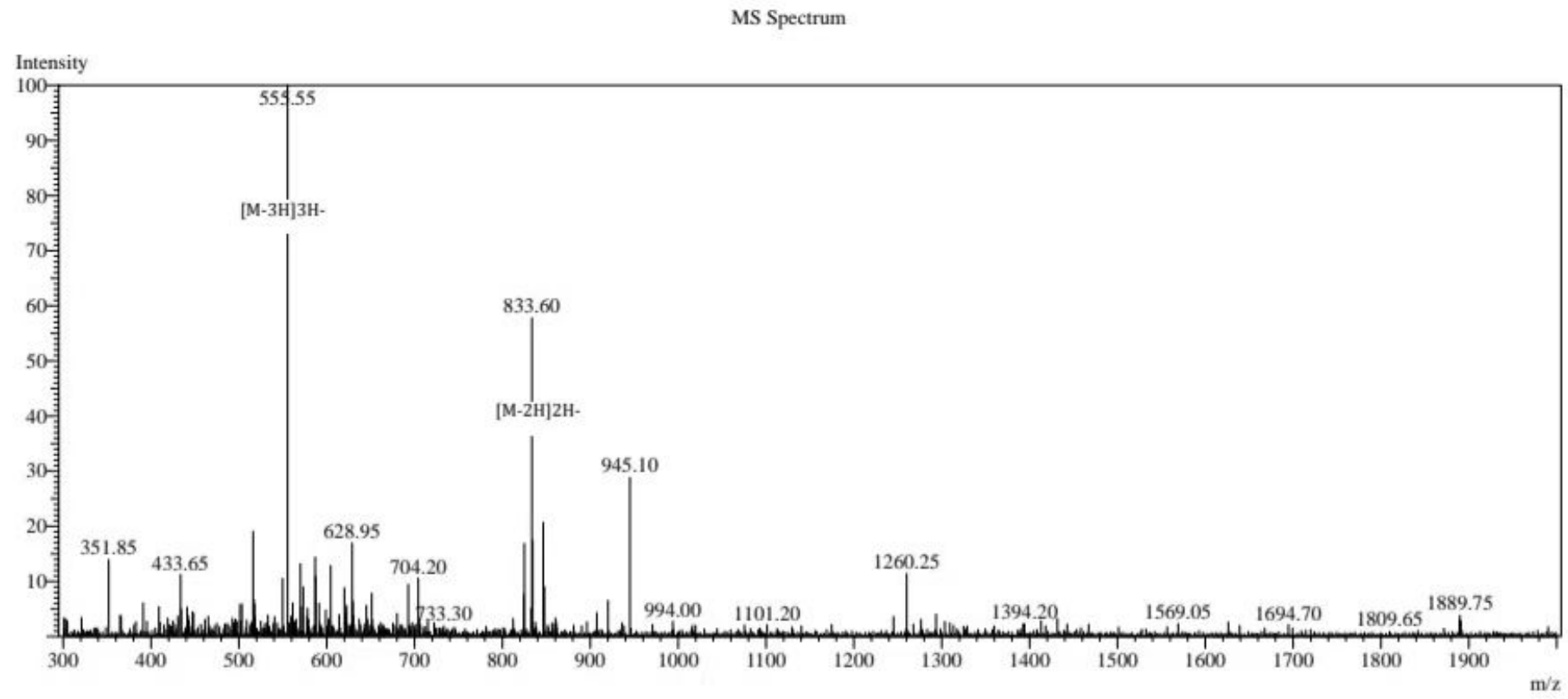

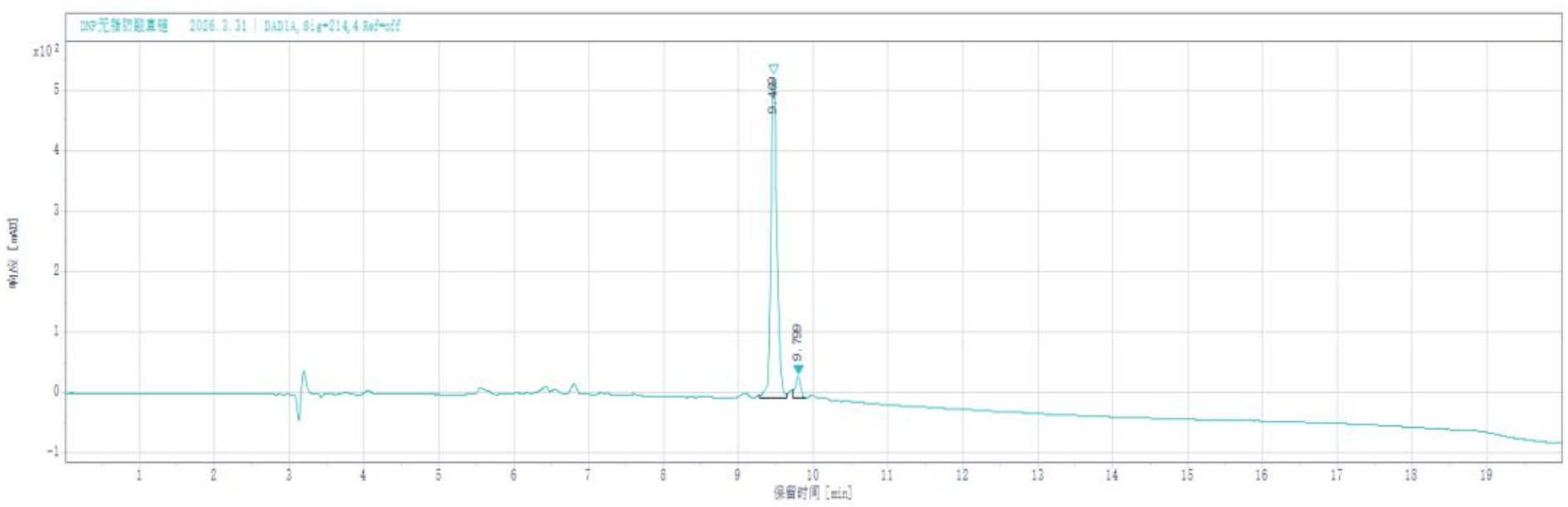

Supplement: Supplementary file 1 [file ao6c05930_si_001.pdf]
